# The tetrapod fauna of the upper Permian Naobaogou Formation of China: 6. *Turfanodon jiufengensis* sp. nov. (Dicynodontia)

**DOI:** 10.7717/peerj.10854

**Published:** 2021-02-17

**Authors:** Jun Liu

**Affiliations:** 1 Key Laboratory of Vertebrate Evolution and Human Origins of Chinese Academy of Sciences, Institute of Vertebrate Paleontology and Paleoanthropology, Chinese Academy of Sciences, Beijing, China; 2 CAS Center for Excellence in Life and Paleoenvironment, Beijing, China; 3 College of Earth and Planetary Sciences, University of Chinese Academy of Sciences, Beijing, China

**Keywords:** Upper Permian, Naobaogou Formation, Dicynodontia, Dicynodontoidea, *Turfanodon*

## Abstract

The dicynodont fossils from the Naobaogou Formation of Nei Mongol, China are abundant and diverse but poorly studied. In this article, one nearly complete skeleton and four cranial specimens from the Naobaogou Formation are referred to the dicynodontoid genus *Turfanodon* as a new species, *T. jiufengensis*. Previously, *Turfanodon* was known only from upper Permian sites in Xinjiang and Gansu. The new specimens are referred to *Turfanodon* based on the following characters: snout tall with steeply sloping profile, anterior tip of the snout squared off, facial region heavily pitted, nasal bosses present as paired swellings near the posterodorsal margin of the external nares, preparietal depressed, intertemporal bar long and narrow, premaxilla contacting frontal, palatal surface of premaxilla exposed in lateral view, and anterior pterygoid keel restricted to the anterior tip of the anterior ramus of the pterygoid. *Turfanodon jiufengensis* is differentiated from the type species, *T. bogdaensis*, by a contact of the lacrimal with the septomaxilla, discrete, raised nasal bosses, the dorsal edge of the erupted portion of the canine tusk slightly posterior to the anterior orbital margin, an anterior extension of the lacrimal distinctly shorter than that of the prefrontal, and a premaxillary dorsal surface with a median ridge. The holotype skeleton of *T*. *jiufengensis* includes a complete axial column with 50 vertebrae (six cervical, 23 dorsal, six sacral, and 15 caudal). *Turfanodon* represents the first confirmed tetrapod genus shared by the late Permian faunas of the Junggar and Ordos basins, and appears to be the first dicynodont genus distributed across both tropical and temperate zones (based on paleoclimate reconstructions). Based on tetrapod fossil content, the Naobaogou Formation can be roughly correlated to the *Daptocephalus* Assemblage Zone of South Africa (255–252 Ma in age).

## Introduction

In 1963–1964, dicynodont specimens were collected from the strata exposed on the northern (Jimsar) and southern (Turpan) flanks of the Bogda Mountains, Xinjiang by a field team from the Institute of Vertebrate Paleontology and Paleoanthropology, Chinese Academy of Sciences (IVPP). One large skull from the Guodikeng Formation of Turpan, IVPP V 3241, was named *Turfanodon bogdaensis*, and diagnosed by “skull rather large, triangular in dorsal view, squamosal extends posterolaterally; a distinct ridge below the lower rim of external naris; snout rather broad and large; orbits dorsolaterally directed, interorbital region wide, temporal fenestra triangular; narrow postfrontal present; frontal with a middle ridge on anterior part, frontal meets premaxilla; intertemporal region not elevated, parietal ridge with a narrow and long middle groove; parietal deepened; interpterygoid vacuity rather small, 1/5 length of basicranial axis” (Sun, 1973, p. 56). Another incomplete, larger skull from the Guodikeng Formation of Jimsar, IVPP V 4,694, was named *Striodon magnus*, and diagnosed as a “very large dicynodont with total skull length greater than 600 mm; occiput low and wide, squamosal laterally extended; large temporal opening roughly square; intertemporal bar long and narrow, pineal foramen large, situated in a depression anterior to intertemporal bar” (Sun, 1978, p. 20).

In 1982, a third large dicynodont skull, IGCAGS V296 (now housed at IVPP), was collected from the Sunan Formation of Lugou, Sunan, Gansu by Cheng Zheng-Wu. It was named *Dicynodon sunanensis*, and this taxon was diagnosed as a “large dicynodont, maximum length of skull reaching 400 mm; posterior end of premaxilla in contact with frontal, which separating two nasals; a deep pit present at premaxilla-frontal suture and a shallow groove extending along midline of premaxilla; nasal bosses developed; lacrimal extending more anteriorly than prefrontal; labial fossa absent” (Li, Cheng & Li, 2000, p. 150). They mentioned that this taxon was most similar to *Turfanodon bogdaensis* (at the time called *Dicynodon bogdaensis*) among known species.

King (1988) considered *Turfanodon* to be a junior synonym of *Dicynodon*, albeit maintaining the type species as valid in a new combination (*D. bogdaensis*), and this opinion was adopted by most subsequent researchers (Li, Wu & Zhang, 2008; Lucas, 1998, 2001). She also suggested that *Striodon magnus* might be referable to *Dicynodon*, but considered the holotype too incomplete to be certain. Lucas (1998, 2001) regarded *Striodon magnus* as a *nomen dubium*, referring the type specimen IVPP V 4694 to *Dicynodon* sp. Kammerer, Angielczyk & Fröbisch (2011) revised the genus *Dicynodon*, revalidating *Turfanodon* as a separate genus but synonymizing *Striodon magnus* and *Dicynodon sunanensis* with *Turfanodon bogdaensis*. If this taxonomic hypothesis is accepted, then *T*. *bogdaensis* is present in the upper Permian of both Xinjiang and Gansu.

*Daqingshanodon limbus* from the Naobaogou Formation was historically the only known Permian dicynodont from the Ordos Basin (Zhu, 1989). However, from 2009 to 2011, many dicynodont specimens were discovered in the Naobaogou Formation of Daqingshan area, Nei Mongol (Liu, 2019). Recently, dicynodonts were also described from the upper Permian Sunjiagou Formation in Shanxi (Liu, 2020; Yi & Liu, 2020). Here, several of the dicynodont specimens from the Naobaogou Formation of Nei Mongol are described in detail, including the first relatively complete dicynodont skeleton from the Chinese upper Permian. This specimen, together with four others, represents a new species of *Turfanodon*. It is the first tetrapod genus shared by the late Permian faunas of the Junggar Basin (Xinjiang) and the Ordos Basin (North China).

### Geological setting

**Figure 1:**
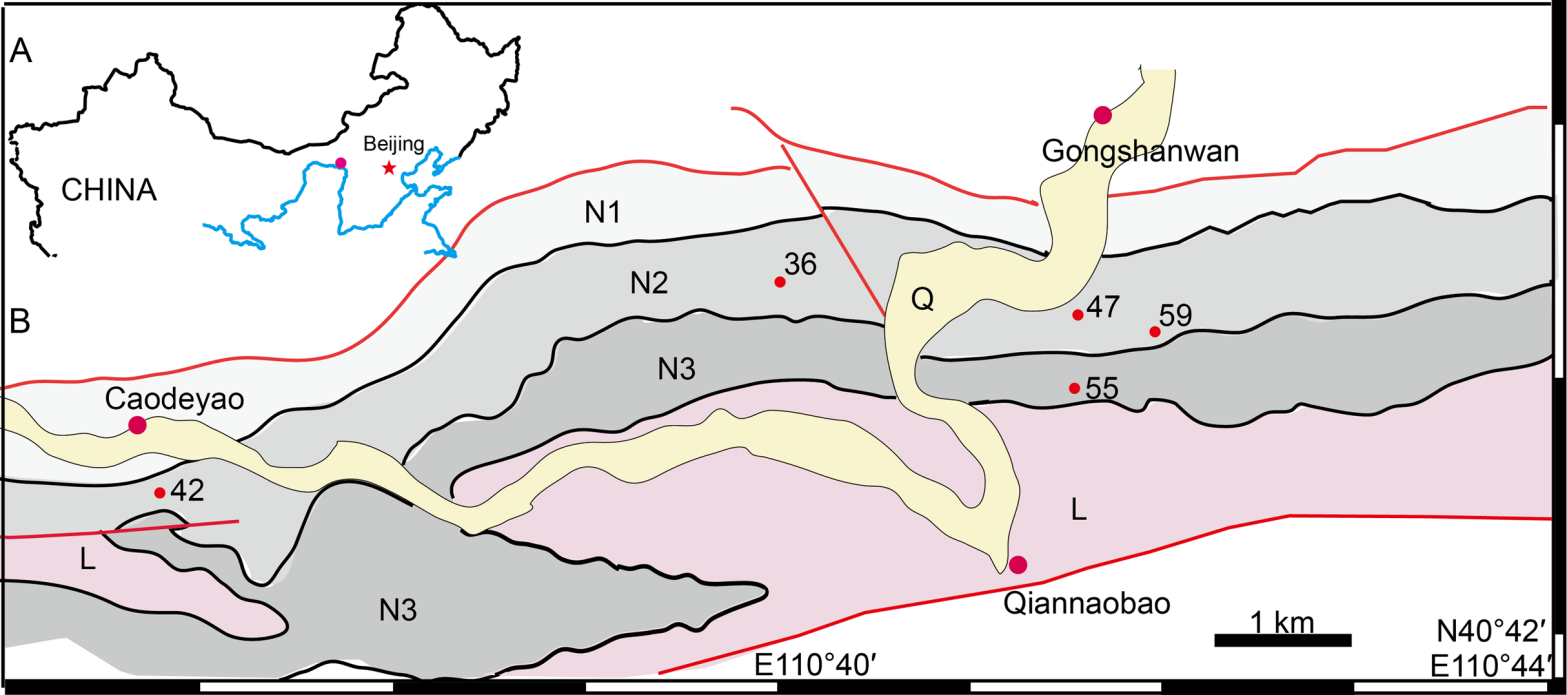
Map of the fossil localities of *Turfanodon jiufengensis*. (A) Geographical location on simplified map (in red circle). (B) Simplified geological map showing fossil localities: 36, IVPP V 26035; 42, IVPP V 23879; 47, IVPP V 23880; 55, IVPP V 26038; 59, IVPP V 23299. Abbreviations: L, Laowopu Formation, N1–3, members I–III of Naobaogou Formation; Q, Quaternary. Drawing credit: Jun Liu.

The geological background of the studied specimens was discussed in previous articles (Liu, 2019; Liu & Abdala, 2017a; Liu & Bever Gabriel, 2018; Zhu, 1989) and will not be repeated here. The fossil localities are distributed in a small area close to Gongshanwan, Tumed Right Banner, Nei Mongol, China (Fig. 1). Most specimens were collected in Member II of the Naobaogou Formation, with only IVPP V 26038 coming from the upper part of Member III (Locality DQS 55).

### Nomenclatural acts

The electronic version of this article in portable document format will represent a published work according to the International Commission on Zoological Nomenclature (ICZN), and hence the new names contained in the electronic version are effectively published under that Code from the electronic edition alone. This published work and the nomenclatural acts it contains have been registered in ZooBank, the online registration system for the ICZN. The ZooBank Life Science Identifiers (LSIDs) can be resolved and the associated information viewed through any standard web browser by appending the LSID to the prefix http://zoobank.org/. The LSID for this publication is: urn:lsid:zoobank.org:pub:72E571BB-4982-4294-886C-4700F9596AA7. The online version of this work is archived and available from the following digital repositories: PeerJ, PubMed Central, and CLOCKSS.

## Systematic Palaeontology

ANOMODONTIA Owen, 1860

DICYNODONTIA Owen, 1860

DICYNODONTOIDEA Olson, 1944

*Turfanodon* Sun, 1973

**Type species.** *Turfanodon bogdaensis* Sun, 1973.

**Revised diagnosis.** Large dicynodontoid with a tall, steeply-sloping snout, heavily pitted facial region, depressed preparietal, and elongate, narrow intertemporal bar. Differentiated from all dicynodontoids other than *Dinanomodon* by a contact between the premaxilla and frontal, palatal surface of premaxilla exposed in lateral view, and anterior pterygoid keel restricted to the anterior tip of the anterior ramus of the pterygoid. Differentiated from *Dinanomodon* by having the anterior tip of the snout squared off and nasal bosses present as anteroposteriorly elongated swellings near the posterodorsal margin of external nares.

*Turfanodon jiufengensis* sp. nov.

**Etymology.** ‘Jiufeng’, meaning ‘nine peaks’, the name of the mountain producing the known specimens.

**Holotype.** IVPP V 26038, a relatively complete skeleton, including an incomplete skull and lower jaw.

**Type locality and horizon.** Locality DQS55, Tumed Right Banner, Nei Mongol, China; Member III, Naobaogou Formation.

**Referred material.** IVPP V 23299, a crushed skull with lower jaw; IVPP V 23879, a well-preserved snout; IVPP V 23880, an incomplete small skull; IVPP V 26035, a three-dimensionally preserved skull, 10 vertebrae, and an incomplete right forelimb. All from Member II, Naobaogou Formation.

**Diagnosis.** A large Permian dicynodontoid with 50 vertebrae (six cervical, 23 dorsal, six sacral, and 15 caudal). Differentiated from its congener *Turfanodon bogdaensis* by a contact between the lacrimal and septomaxilla (separating the maxilla from the prefrontal), well-developed and distinctly raised nasal bosses, dorsal edge of the erupted portion of the canine tusk slightly posterior to the anterior orbital margin, anterior extension of lacrimal distinctly shorter than that of the prefrontal, and a preparietal with an anterolateral processes.

### Description

**Figure 2:**
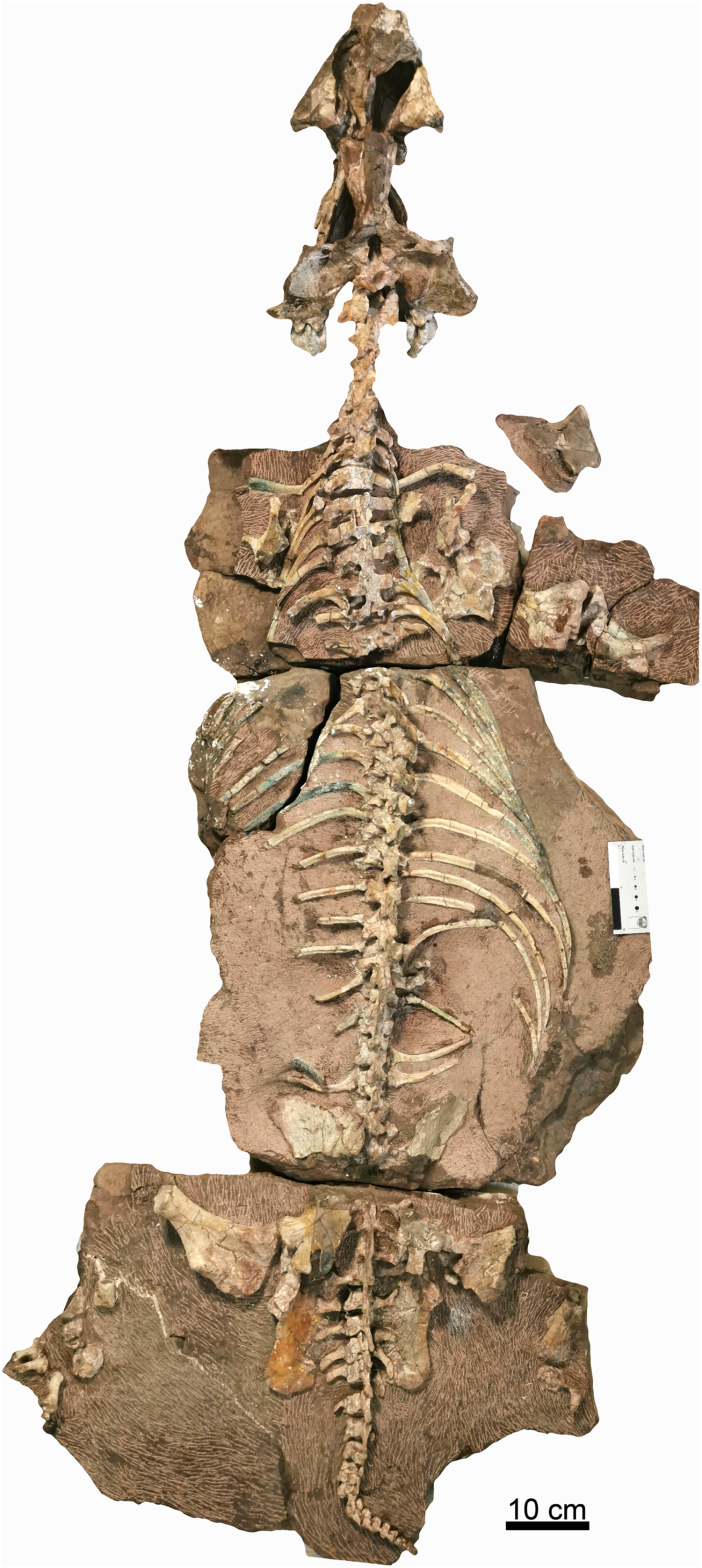
Holotype of *Turfanodon jiufengensis* (IVPP V 26038) from the Naobaogou Formation. Skeleton in dorsal view. Photo credit: Jun Liu.

**Figure 3:**
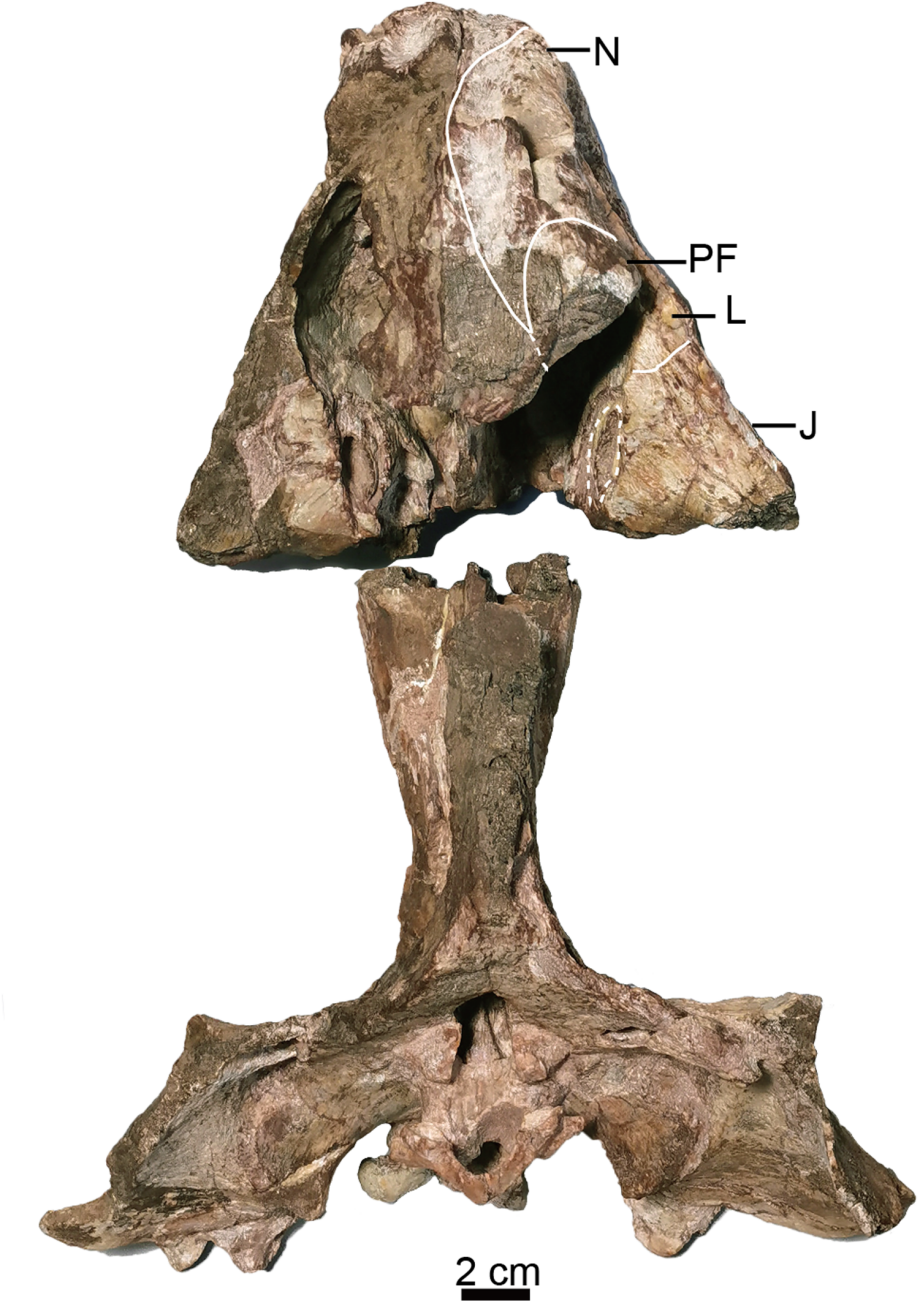
Holotype of *Turfanodon jiufengensis* (IVPP V 26038) from the Naobaogou Formation. Skull in dorsal view. Abbreviations: J, jugal; L, lacrimal; N, nasal; PF, prefrontal. Photo credit: Jun Liu.

**Figure 4:**
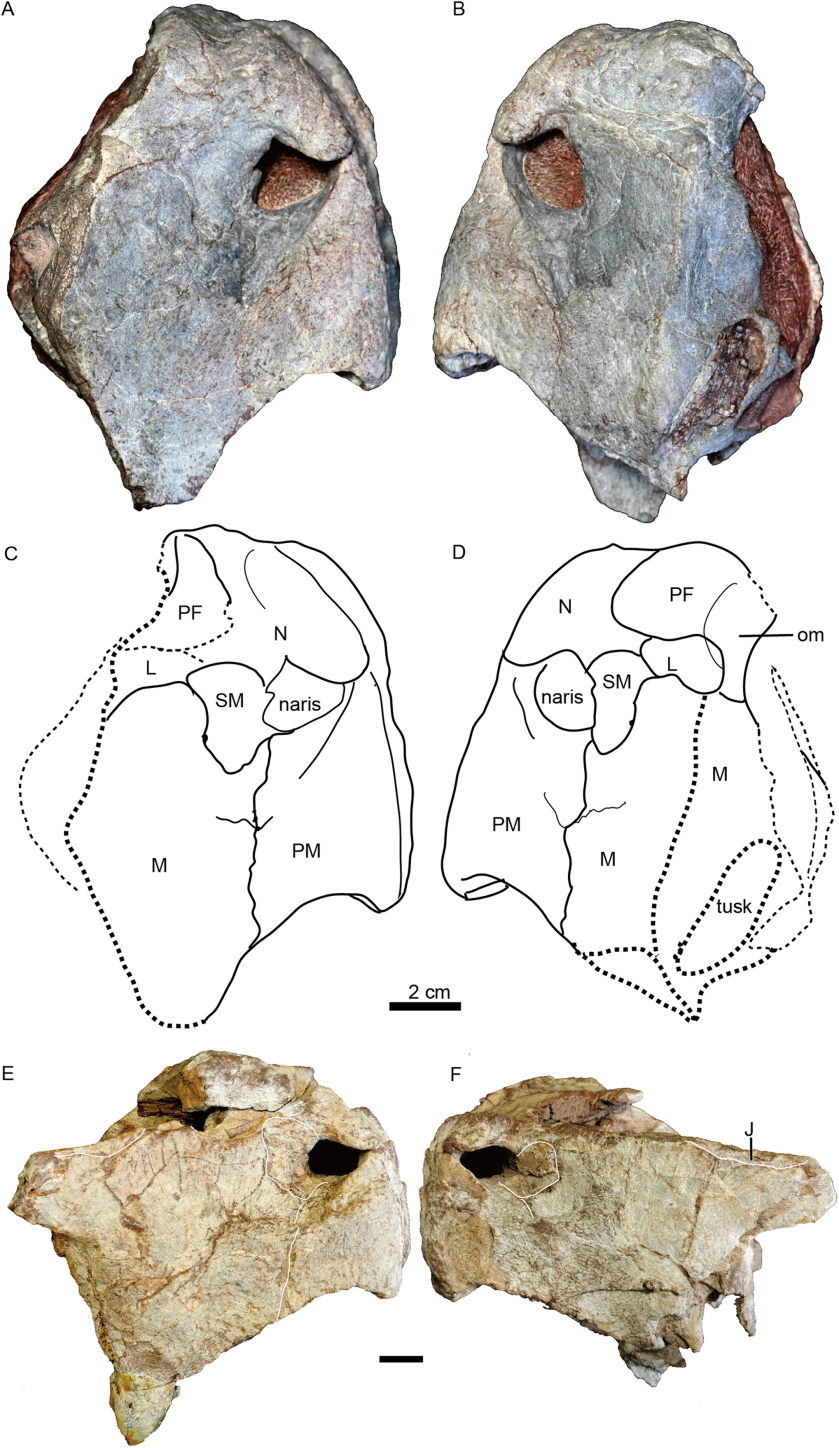
*Turfanodon jiufengensis* from the Naobaogou Formation, snout in lateral view. ****IVPP V 23879, photos in (A) right and (B) left lateral views, drawings in (C) right and (D) left lateral views. Holotype, IVPP V 26038, photos in (E) right and (F) left lateral views. Abbreviations: J, jugal; L, lacrimal; M, maxilla; N, nasal; om, orbital margin; PF, prefrontal; PM, premaxilla; SM, septomaxilla. Scale bars equal 2 cm. Photo/drawing credit: Jun Liu.

**Figure 5:**
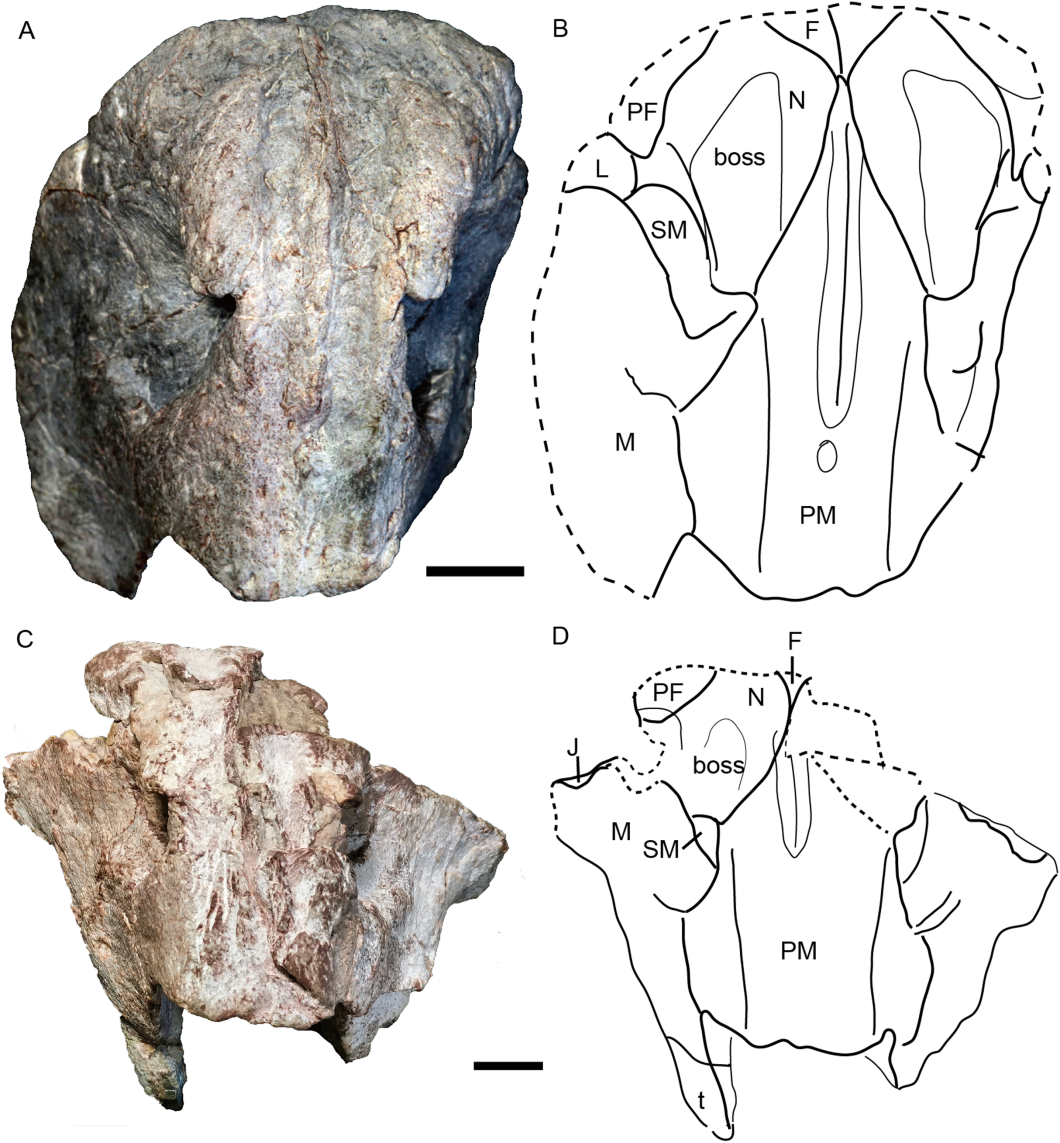
*Turfanodon jiufengensis* from the Naobaogou Formation, snout in anterior view. (A) Photo and (B) drawing of IVPP V 23879; (C) photo and (D) drawing of holotype (IVPP V 26038). Abbreviations: F, frontal; J, jugal; L, lacrimal; M, maxilla; N, nasal; PF, prefrontal; PM, premaxilla; SM, septomaxilla. Scale bars equal 2 cm. Photo/drawing credit: Jun Liu.

**Figure 6:**
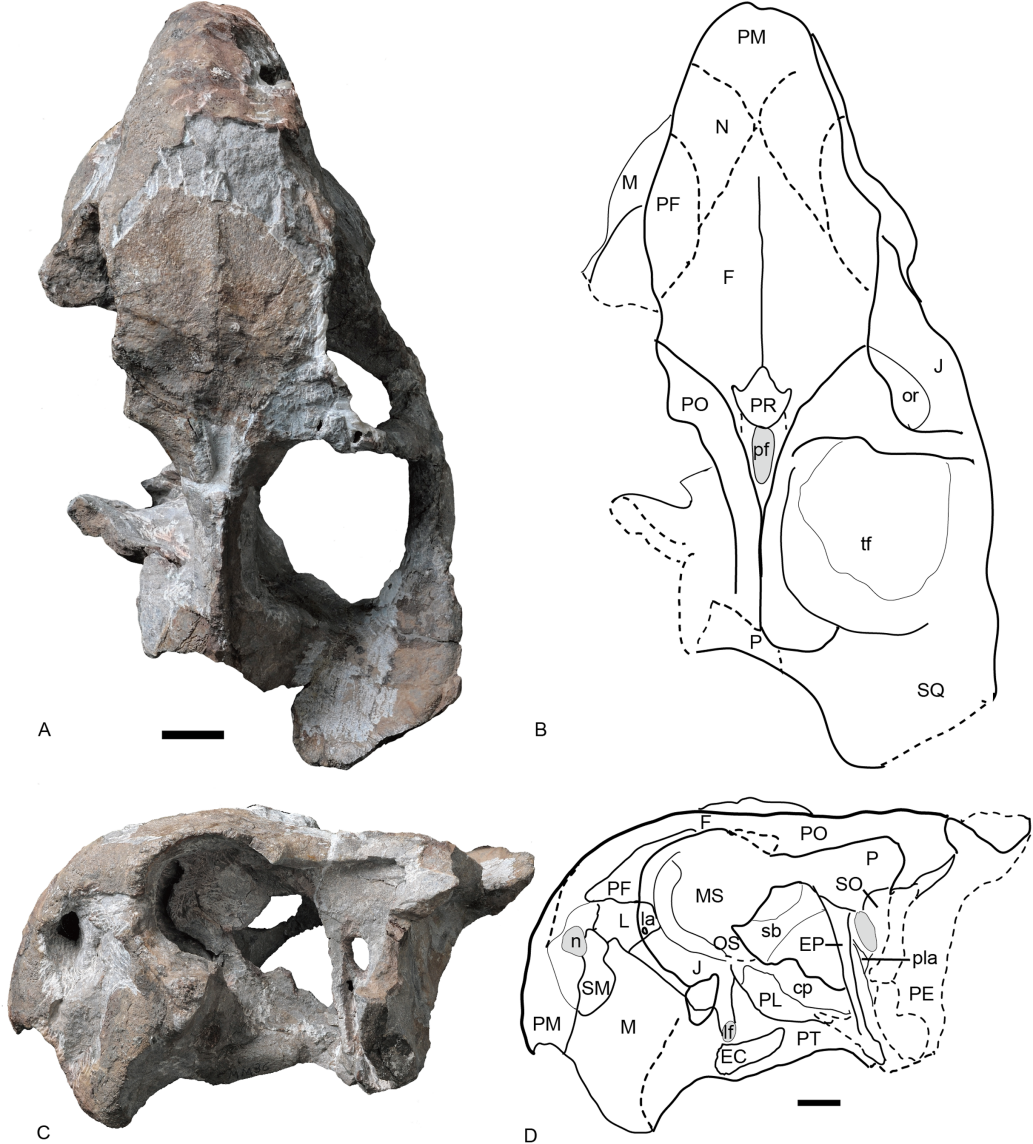
Referred specimen of *Turfanodon jiufengensis* (IVPP V 26035) from the Naobaogou Formation. (A) Photo and (B) drawing of skull in dorsal view; (C) photo and (D) drawing of skull in left lateral view. Abbreviations: cp, cultriform process of parasphenoid; EC, ectopterygoid; EP, epipterygoid; F, frontal; J, jugal; L, lacrimal; la, lacrimal fossa; lf, labial fossa; M, maxilla; MS, mesethmoid; n, naris; N, nasal; or, orbit; OS, orbitosphenoid; PE, periotic; pf, pineal foramen; PF, prefrontal; PL, palatine; pla, pila antotica; PM, premaxilla; PO, postorbital; PP, postparietal; PR, preparietal; sb, suborbital bar; SM, septomaxilla; SO, supraoccipital; SQ, squamosal; tf, temporal fenestra. Scale bars equal 2 cm. Photo credit: Wei Gao; drawing credit: Jun Liu.

**Figure 7:**
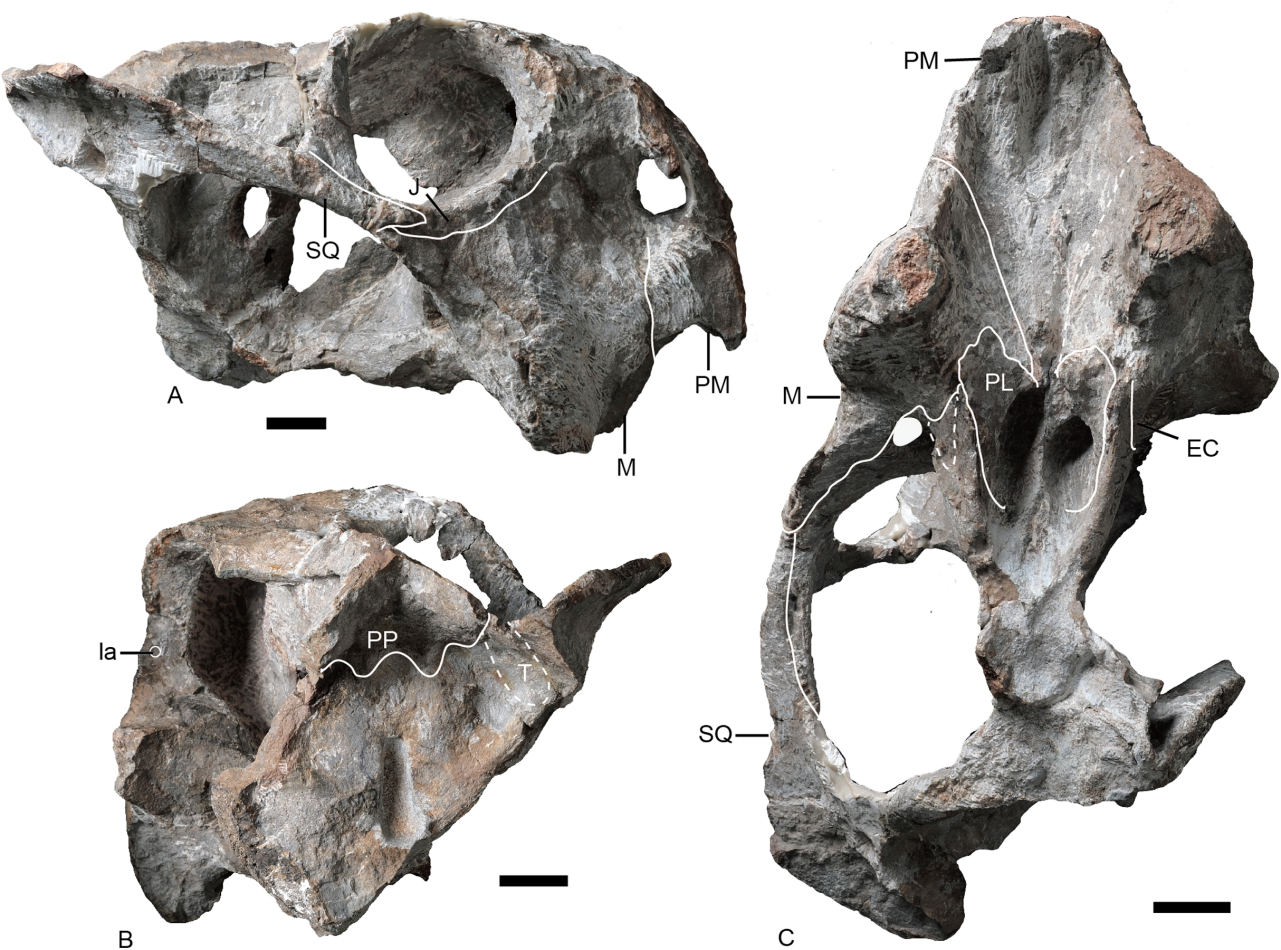
Referred specimen of *Turfanodon jiufengensis* (IVPP V 26035) from the Naobaogou Formation. ****Skull in (A) right lateral, (B) posterior, and (C) ventral views. Scale bars equal 2 cm. Abbreviations: EC, ectopterygoid; J, jugal; la, lacrimal fossa; M, maxilla; PL, palatine; PM, premaxilla; PP, postparietal; SQ, squamosal; T, tabular. Photo credit: Wei Gao; drawing credit: Jun Liu.

**Figure 8:**
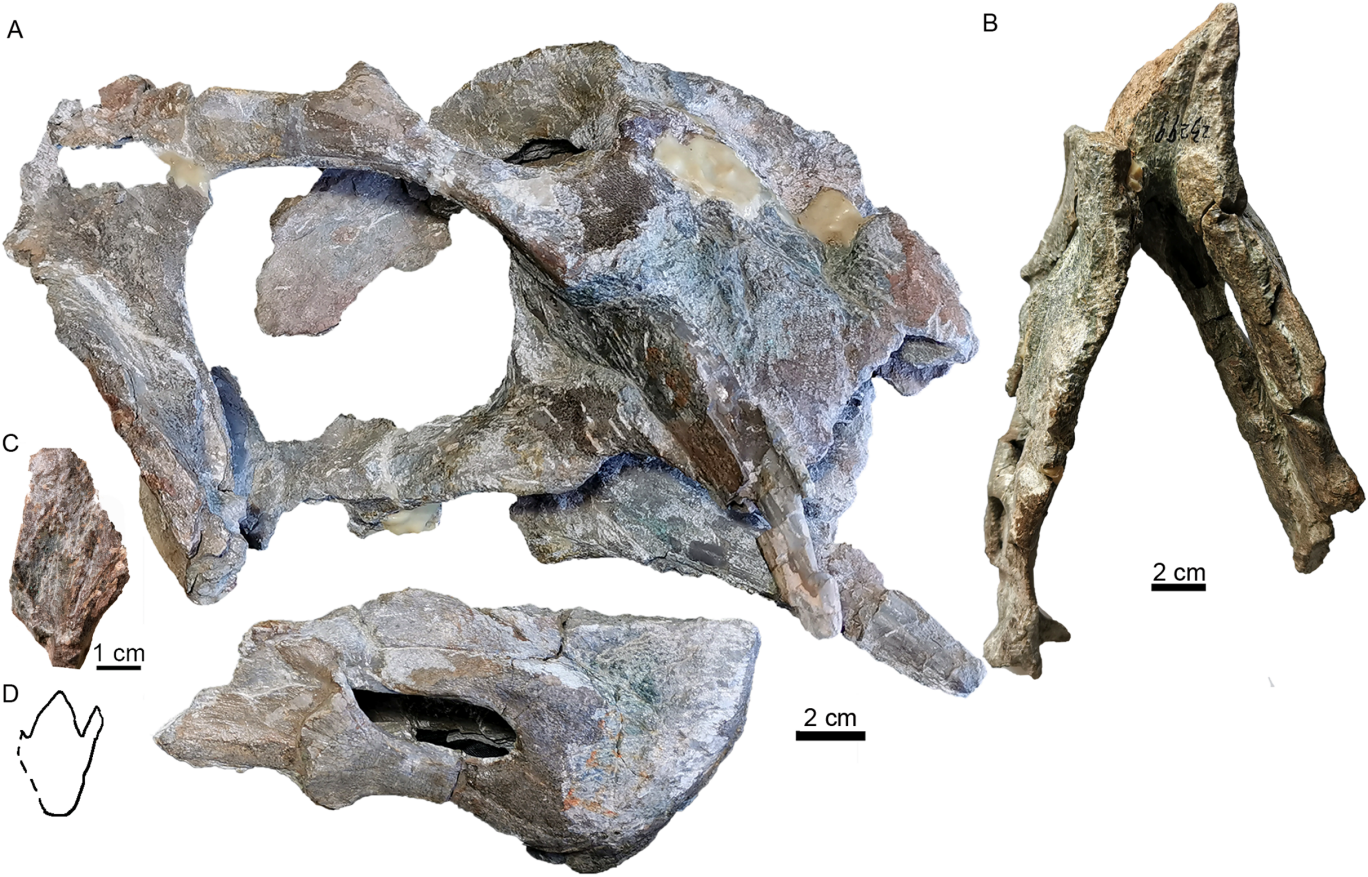
Referred specimen of *Turfanodon jiufengensis* (IVPP V 23299) from the Naobaogou Formation. (A) Skull and lower jaw in right lateral view; (B) lower jaw in dorsal view; (C) photo and (D) drawing of preparietal in dorsal view. Photo/drawing credit: Jun Liu.

**Figure 9:**
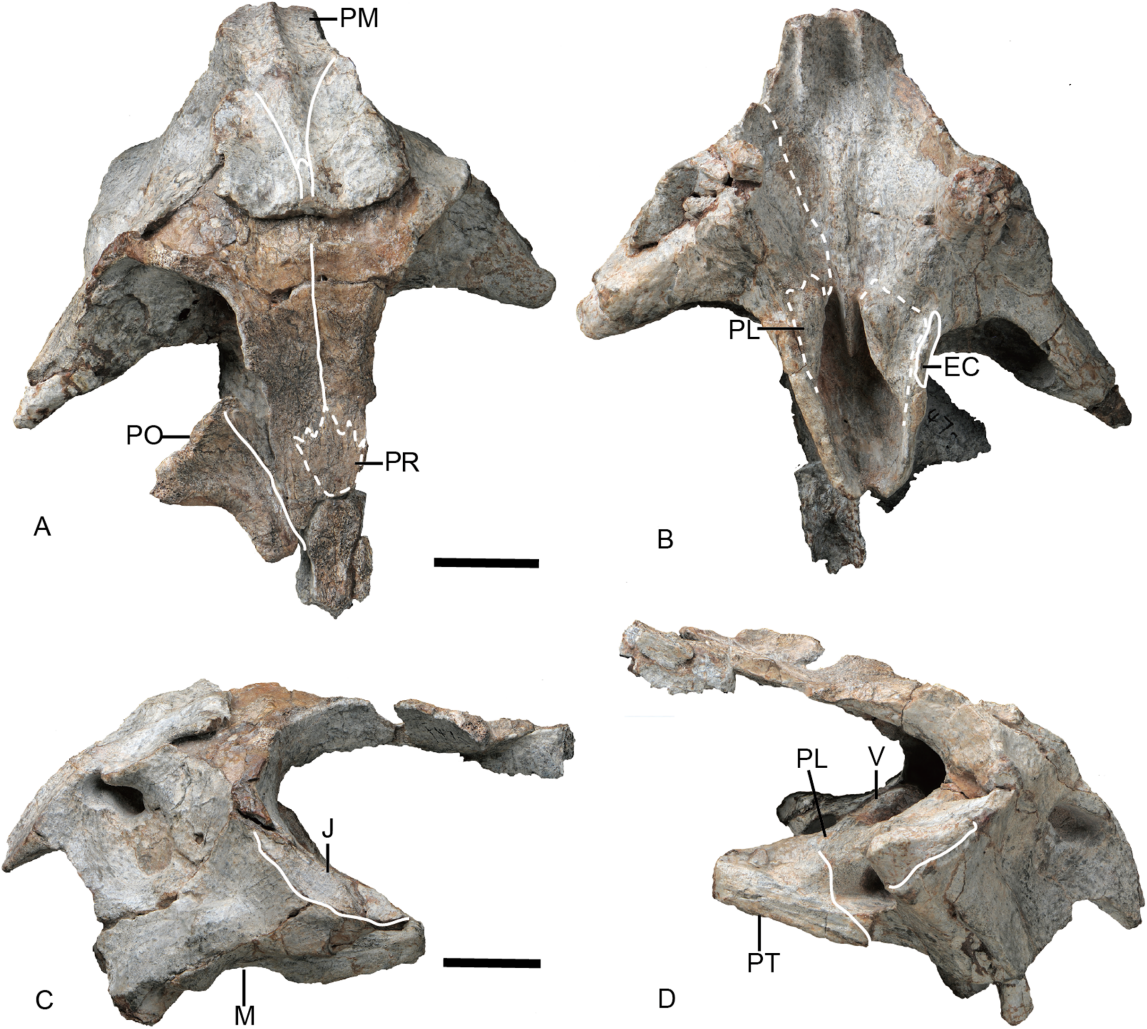
Referred specimen of *Turfanodon jiufengensis* (IVPP V 23880) from the Naobaogou Formation. Skull in (A) dorsal, (B) ventral, (C) left lateral, and (D) right lateral views. Abbreviations: EC, ectopterygoid; J, jugal; M, maxilla; PL, palatine; PM, premaxilla; PO, postorbital; PR, preparietal; PT, pterygoid; V, vomer. Scale bars equal 2 cm. Photo credit: Wei Gao.

The holotype (IVPP V 26038) is a relatively complete skeleton with a total body length of ~180 cm (Fig. 2). Its skull was eroded into a relatively complete anterior part and an incomplete posterior part with a nearly complete palatal complex and braincase, and there is a short gap between the two portions (Fig. 3). This skull measures ~33 cm from the tip of the snout to the occipital condyle. Another similarly-sized specimen, IVPP V 23879, preserves only the snout, but the sutures can be more clearly observed (Figs. 4A–4D, 5A and 5B). IVPP V 23299 and 26035 are smaller in size (Table 1), with the latter being the better-preserved of the two, but with less clear sutures than IVPP V 23879 (Figs. 6–8). The smallest specimen, IVPP V 23880, consists of a partial skull preserving the snout and part of the orbital region (Fig. 9). The following description is mainly based on two larger specimens (IVPP V 26038 and 23879) unless otherwise specified.

**Table 1:** Cranial measurements (in cm) and some cranial features of all referred specimens of *Turfanodon*.

	IVPP V 3241	IGCAGS 296	IVPP V 4694	IVPP V 23880	IVPP V 26035	IVPP V 23299	IVPP V 23879	IVPP V 26038
BLS	~31	37	>60	~13	20	~22	?	33
OW	29	31	56	?		?	?	~26
OH	18	~17	24	?	?	~13	?	~16
C1	A	A	?	P	?	?	P	P
C2	A	P	?	P	?	?	P	P
C3	P	P	?	V	V	?	V	V
C4	P	A	?	A	A	A	A	A
C5	?	A	?	P	P	?	P	P
C6	A	A	?	?	P	?	P	P
C7	?	W	?	W	W	W	?	N
C8	A	A	?	?	P	P	?	?
C9	G	G	G	?	C	?	?	?
C10	H	H	R	?	H	?	?	H

**Note:**

BLS, basal length of skull; OW, occipital width; OH, occipital height; A, absent; P, present. C1, premaxillary medial ridge; C2, nasal boss on dorsal margin of naris; C3, dorsal tip of premaxilla where it meets frontal: (P) forming a pit, (V) a low but convex surface; C4, distinct ridge below the naris on lateral surface of maxilla; C5, septomaxilla contact lacrimal; C6, prefrontal extension anteriorly relative to lacrimal; C7, jugal lateral exposure: (W) wide, (N) narrow; C8, preparietal anterolateral processes; C9, parietal exposed on skull roof: (G) groove, (C) narrow and crest-like; C10, foramen magnum: (H) height to width greater than 2; (R) rounded.

### Skull

**Figure 10:**
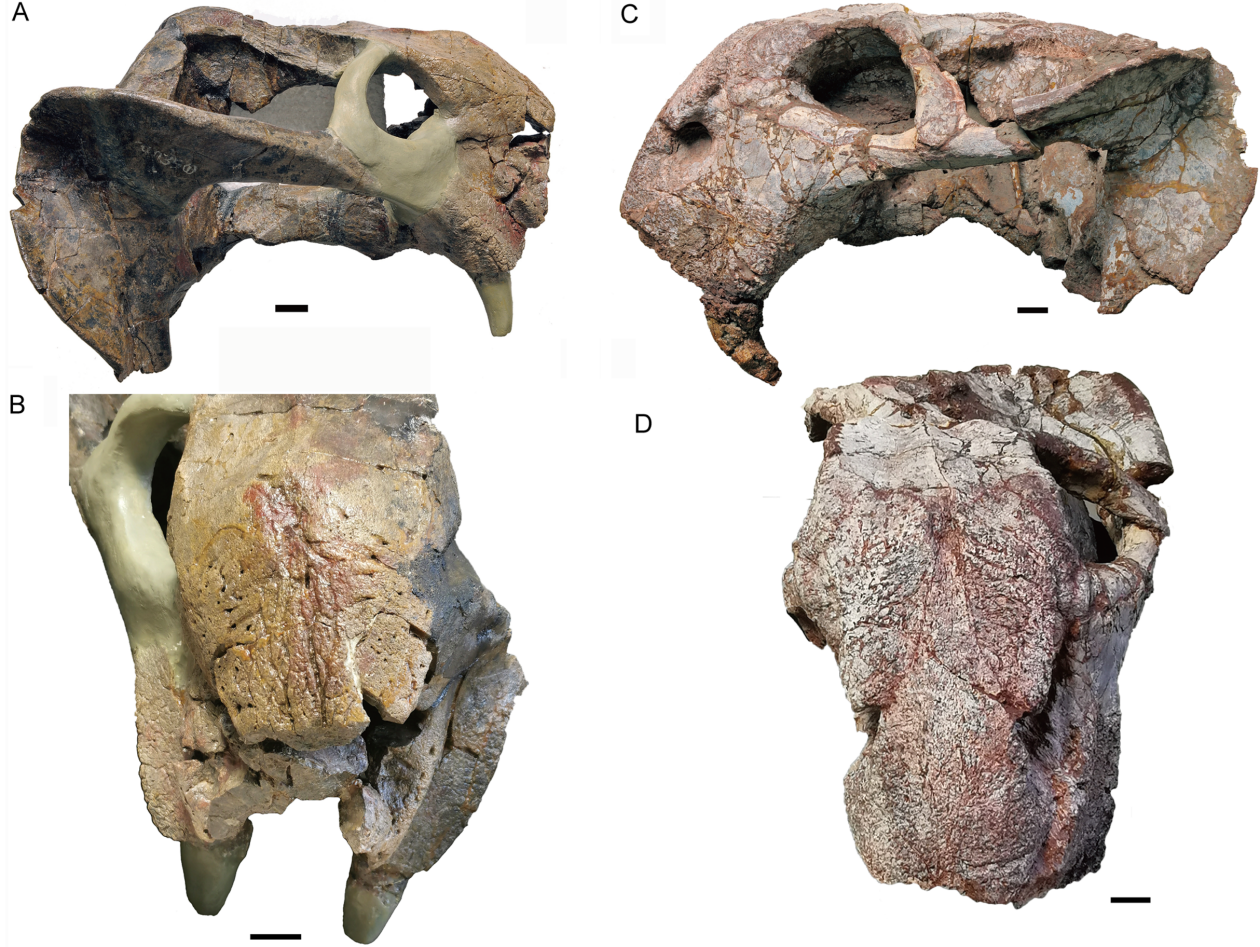
Skull of *Turfanodon bogdaensis*. Holotype (IVPP V 3241) in (A) right lateral and (B) anterodorsal views. IGCAGS V296 (holotype of *Dicynodon sunanensis*) in (C) left lateral and (D) anterodorsal views. Scale bars equal 2 cm. Photo credit: Jun Liu.

**Figure 11:**
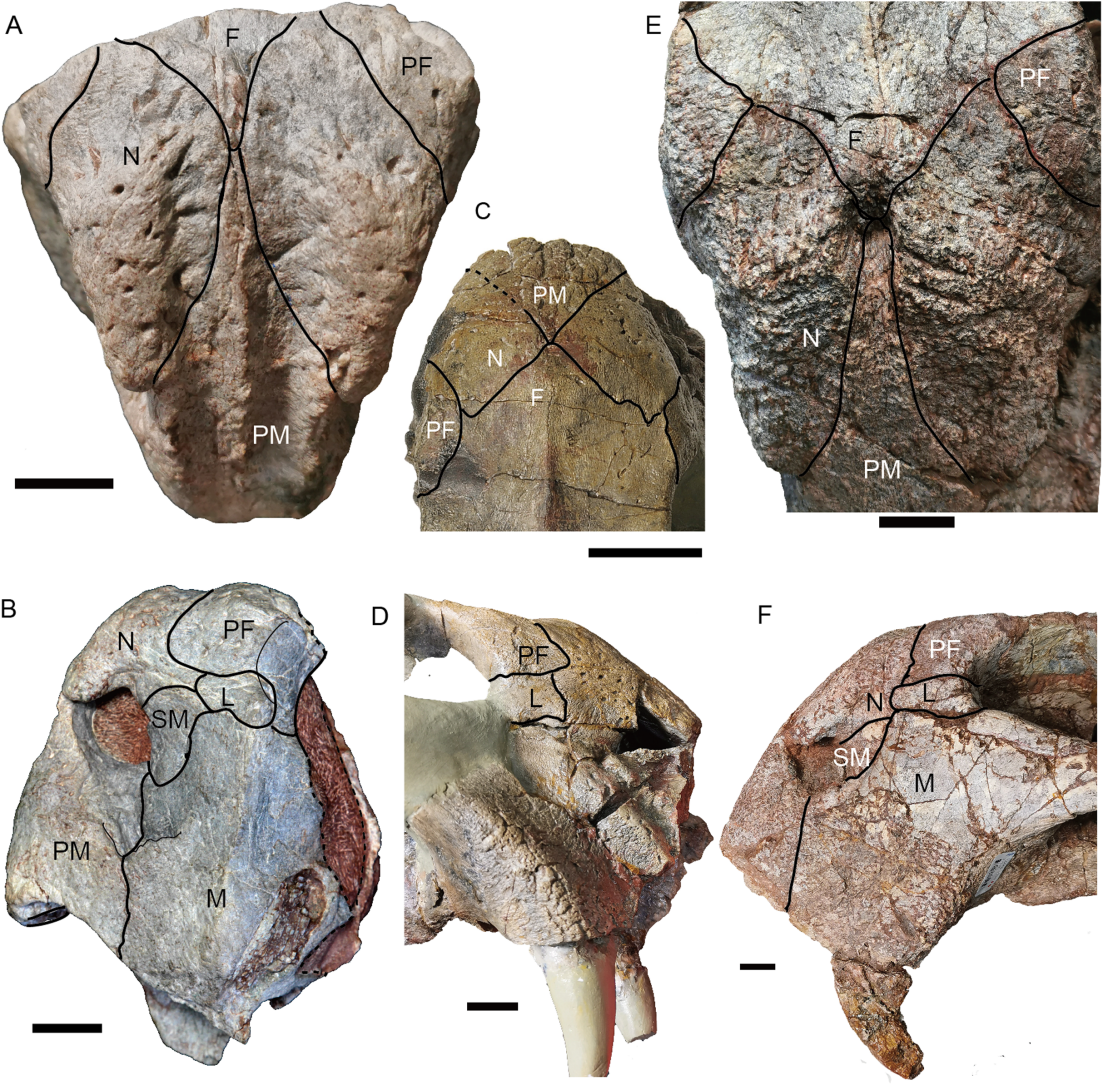
Comparison of skulls of *Turfanodon jiufengensis* and *T. bogdaensis* in dorsal (A, C and E) and lateral views (B, D and F). *Turfanodon jiufengensis* (A and B) IVPP V 23879; *T. bogdaensis*(C and D) IVPP V 3241 (E and F) IGCAGS V296 (holotype of *Dicynodon sunanensis*). Abbreviations: F, frontal; J, jugal; L, lacrimal; M, maxilla; N, nasal; PF, prefrontal; PM, premaxilla; SM, septomaxilla. Scale bars equal 2 cm. Photo/drawing credit: Jun Liu.

The snout is sharply sloping and tall (Figs. 4–9), as in *Turfanodon bogdaensis*, *Peramodon amalitzkii*, and *Daptocephalus* spp. (Kammerer, 2019; Kammerer, Angielczyk & Fröbisch, 2011). The ventral portion of the anterior premaxillary surface is nearly vertical, as in *P. amalitzkii* (Kurkin, 2012), when the long axis of the skull is aligned to the horizontal. The one exception is in the small skull IVPP V 23880, in which the snout profile is more weakly angled, like that of *T. bogdaensis* (Figs. 4–11).

The external naris is ~2 cm in diameter, with a large embayment ventral and posterior to the opening on the lateral snout surface formed by the premaxilla, septomaxilla, and maxilla (Fig. 4). This embayment has no clear border posteriorly, but has a sharp anterior margin on the premaxilla and a sharp ventral margin on the maxilla. The naris lies on the upper half of the snout, in a relatively higher position than in *Turfanodon bogdaensis* (Figs. 10 and 11).

The premaxilla has a sculptured surface with many different-sized pits, indicating the presence of keratinous beak covering in life. It is a fused median element with a sharp triangular dorsal tip which contacts the frontal above the nares (Figs. 5, 6 and 9). The lateral surface is clearly demarcated from the anterior surface in most specimens (Figs. 5 and 9), but seems uniformly rounded in IVPP V 26035, as is also the case in *Turfanodon bogdaensis* (Figs. 6 and 10). On the lateral surface, the suture between the premaxilla and maxilla runs ventrally from the narial opening within the embayment to the ventral margin of the beak (Figs. 4, 6, 8 and 9). On the anterior premaxillary surface, a median ridge extends dorsally from around the level of the base of the external nares, except in IVPP V 23880 in which the ridge extends from the tip of the premaxilla (Figs. 5 and 9). Two lateral grooves run along the median ridge in IVPP V 23879, 23880, and 26038 (Figs. 5 and 9), but they seem absent or poorly defined in IVPP V 23299 and 26035 (Figs. 6 and 8). In *T. bogdaensis* (based on IGCAGS V296), the premaxillary anterior surface is almost flat except for a shallow median depression dorsally, near its contact with the nasals and the frontals (Figs. 10 and 11).

**Figure 12:**
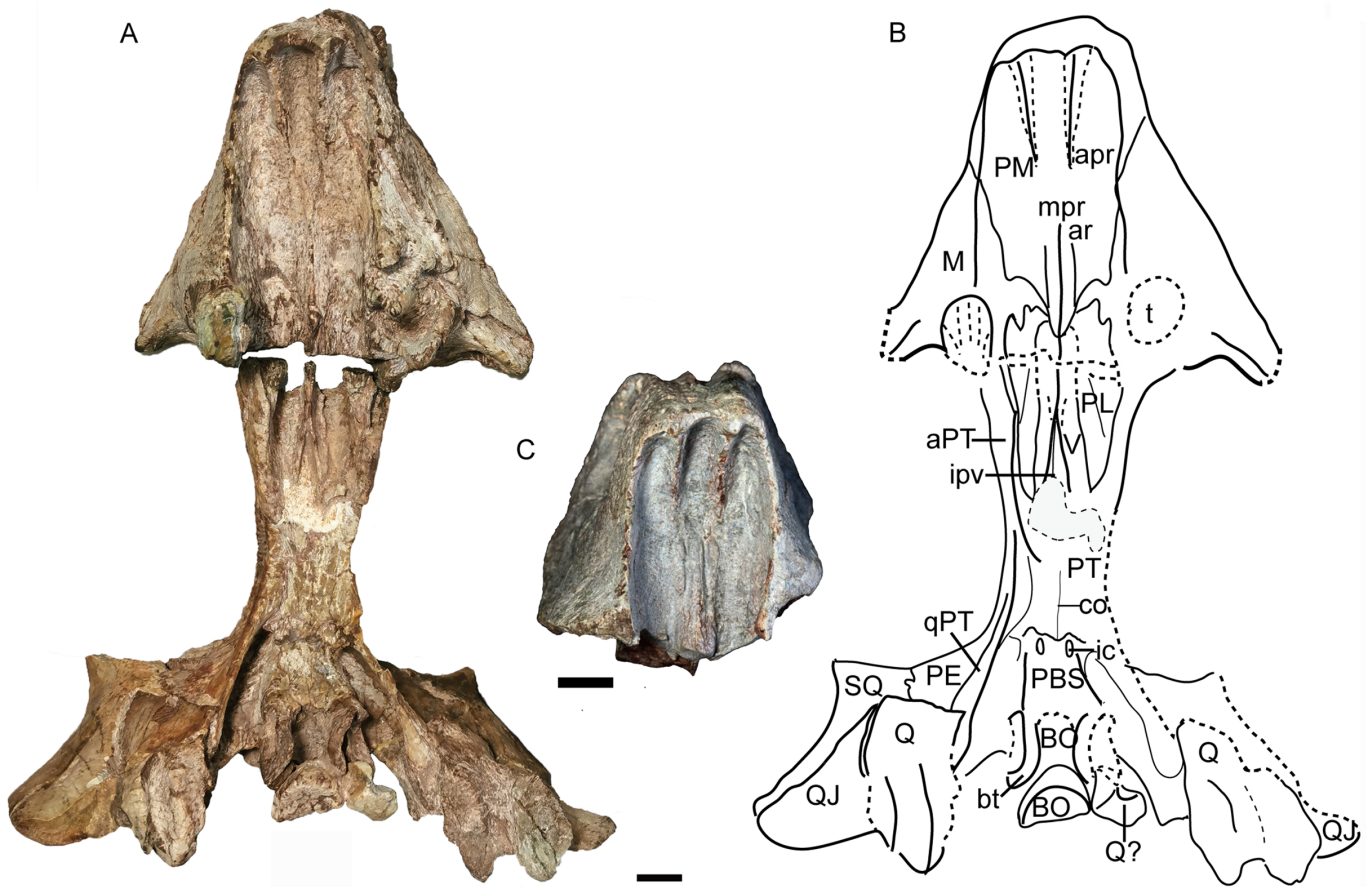
*Turfanodon jiufengensis* from the Naobaogou Formation, skull in ventral view. (A) Photo and (B) drawing of holotype (IVPP V 26038); (C) photo of IVPP V 23879. Abbreviations: apr, anterior palatal ridge; aPT, anterior ramus of the pterygoid; ar, accessory ridge; BO, basioccipital; bt, basal tuber; co, crista oesophagea; ic, internal carotid canal; ipv, interpterygoid vacuity; M, maxilla; mpr, median palatal ridge; PBS, parabasisphenoid; PE, periotic; PL, palatine; PM, premaxilla; PT. pterygoid; Q, quadrate; QJ, quadratojugal; qPT, quadrate ramus of pterygoid; SQ, squamosal; t, tusk; V, vomer. Scale bars equal 2 cm. Photo/drawing credit: Jun Liu.

Ventrally, the premaxilla makes up a broad secondary palatal plate which extends posterior to the level of the tusks and contacts the palatines and vomer (Figs. 7, 9 and 12). Anteriorly, the parallel anterior palatal ridges are sharp and well-developed, such that they can be observed in lateral view (Figs. 4, 7–9). Posterior to them, the medial palatal ridge increases in height posteriorly. Lateral to the median palatal ridge, a pair of accessory ridges runs posteriorly and converges with the median ridge on the posterior part of the premaxilla. These accessory ridges are more distinct in IVPP V 23879 than in IVPP V 26038 (Fig. 12), and are absent in IVPP V 23880 (Fig. 9B).

**Figure 13:**
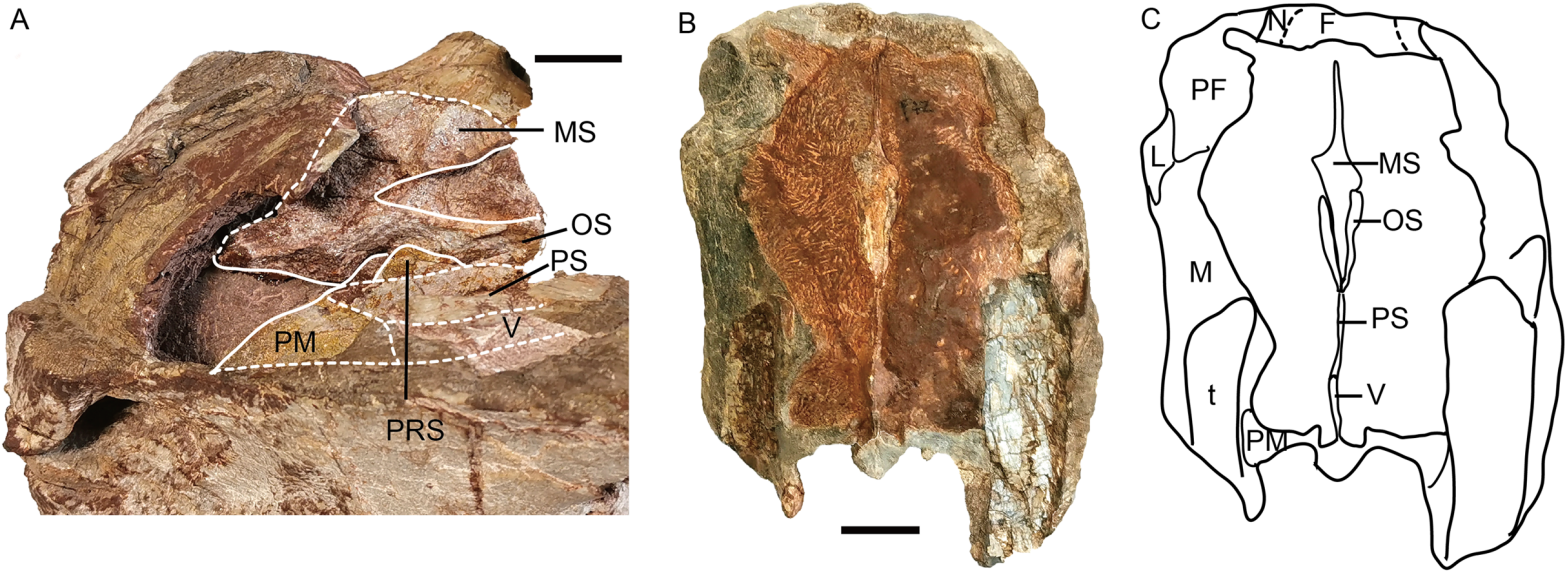
*Turfanodon jiufengensis* from the Naobaogou Formation, snout. Holotype (IVPP V 26038): (A) Photo in left dorsolateral view with interpretive drawing of interior structure. IVPP V 23879: (B) photo and (C) drawing of transverse cross section. Abbreviations: F, frontal; L, lacrimal; M, maxilla; MS, mesethmoid; N, nasal; OS, orbitosphenoid; PF, prefrontal; PM, premaxilla; PRS, presphenoid; PS, parasphenoid; t, tusk, V, vomer. Scale bars equal 2 cm. Photo/drawing credit: Jun Liu.

Anterior to the caniniform tooth, the ventral margin of the premaxilla forms a sharp edge which is almost straight and nearly posteriorly directed in ventral view in large specimens (Fig. 12), slightly laterally angled in the medium-sized IVPP V 26035 (Fig. 7), and strongly laterally angled in the small specimen IVPP V 23880 (Fig. 9). In lateral view, the ventral margin of the premaxilla (forming the cutting edge of the beak) is concave dorsally (Figs. 4, 6–9), as in *Dinanomodon* (Broom, 1938) and *Daptocephalus* (Kammerer, 2019). Dorsal to the palatal plate, the median ridge of the premaxilla contacts the vomer posteriorly and may contact the cultriform process of the parasphenoid dorsally (Fig. 13A).

The septomaxilla is a plate-like bone posterior to the narial opening (Figs. 4 and 6). It has an extensive contact with the maxilla ventrally and posteriorly, but only a short suture with the premaxilla anteroventrally. Dorsally, it contacts the nasal. The posterodorsal corner of the bone contacts the lacrimal. In the holotype of *Turfanodon bogdaensis*, the septomaxilla cannot be identified, but in IGCAGS V296, the septomaxilla approaches but does not contact the lacrimal (Figs. 10 and 11F).

The lateral surface of the maxilla is nearly smooth dorsally, and though rugose ventrally (on and near the caniniform process), this rugosity is more weakly developed than that of the premaxilla (Fig. 4). There is not a distinct, posterodorsal-to-anteroventrally-oriented ridge directly below the external naris as in the holotype of *Turfanodon bogdaensis* (IVPP V 2371) (Figs. 10A and 11D). Ventrally, the maxilla forms a caniniform process housing the tusks. The tusks are anteroventrally directed in IVPP V 23299 (Fig. 8), but are nearly vertical in the undeformed specimens IVPP V 26035 and 26038 (Figs. 4, 7). Anteromedially, a dorsal projection of the maxilla nearly contacts the external naris, almost separating the septomaxilla from the premaxilla. The posterior surfaces of the caniniform processes are slightly concave, based on the condition in IVPP V 26035 (Fig. 7C). The posterior process of the maxilla extends into the zygomatic arch below the jugal and contacts the squamosal just anterior to the postorbital bar (Fig. 7).

The nasals mainly make up the dorsal surface of the snout between the external nares (Figs. 4–9). Each bears a distinct, anteroposteriorly elongate boss on the dorsal margin of the external naris. The edge of the boss extends toward the naris as a pointed projection, giving the dorsal narial margin a ‘notched’ appearance (Fig. 4). The surface of the boss is rugose and densely covered in foramina, as in *T*. *bogdaensis*, but unlike that species is also distinctly raised above the snout surface (Fig. 4). This morphology appears to be distinctive for *T*. *jiufengensis*, although its presence in some specimens (IVPP V 23299 and 26035) is uncertain due to erosion. In IVPP V 23299, small but distinct bosses lie on the posterodorsal corner of the external nares, though the ‘notch’ is absent above the right naris (Fig. 9). In IVPP V 26035, no distinct boss can be discerned, but the corresponding area is rugose (Fig. 6A), suggesting that a thickened covering was present when intact.

The nasal contacts the dorsal extension of the septomaxilla ventrally, and the anterior margin of the lacrimal posteroventrally; so it is separated from the maxilla (Figs. 4–6 and 11B), unlike in *Turfanodon bogdaensis* (Fig. 11F) (Li, Cheng & Li, 2000; Sun, 1973), *Dinanomodon* (Broom, 1938), *Daptocephalus* (Kammerer, 2019), or *Peramodon* (Angielczyk & Kurkin, 2003). The two nasals are completely separated by the contact between the premaxilla with the frontals, similar to the contact in IGCAGS V296, as opposed to the more restricted point contact in the holotype of *T. bogdaensis* (Figs. 5, 9 and 11C). Dorsally, the nasal bears a triangular posterior process extending between the prefrontal and frontal (Figs. 3, 6 and 11A).

The lacrimal is only partially preserved in the two larger specimens (Figs. 3 and 4), but is well preserved in IVPP V 26035 (Figs. 6 and 7). It lies between the prefrontal and the maxilla and contacts the septomaxilla anteriorly. The anterior extension of the bone is distinctly shorter than that of the prefrontal. This is the same as in *Peramodon amalitzkii* (Angielczyk & Kurkin, 2003) and *Daptocephalus huenei* (Kammerer, 2019), but different from *Turfanodon bogdaensis* (Figs. 10 and 11). The lacrimal extends anteriorly at the similar level as the prefrontal in IVPP V 2371 (Sun, 1973), and is longer than it in IGCAGS V296 (Li, Cheng & Li, 2000) (Figs. 11D and 11F). A large lacrimal foramen lies on the posterior face of the lacrimal within the orbit. Medially, the lacrimal is partially covered by the prefrontal and only has a limit exposure on the orbital anterior margin in IVPP V 23879 (Fig. 4); the lacrimal exposure in the orbital margin is relatively broader in IVPP V 26035 (Figs. 6 and 7).

Only the anterior, suborbital portion of the jugal is preserved in the holotype (Figs. 4E and 4F), but it is complete in the two medium-sized specimens IVPP V 23299 and 26035 (Figs. 7 and 8). The suborbital portion of the jugal is mostly exposed dorsally and only exposed laterally as a narrow strip in the holotype, but it has a wide lateral exposure in three smaller specimens (IVPP V 23299, 23880, and 26035) (Figs. 6–9), similar to *Turfanodon bogdaensis* (Figs. 10B and 11F) (Sun, 1973, fig. 3). The jugal is nearly triangular in posteroventral view. It inserts between the maxilla and palatine and contributes to the large labial fossa ventrolaterally (Fig. 7).

**Figure 14:**
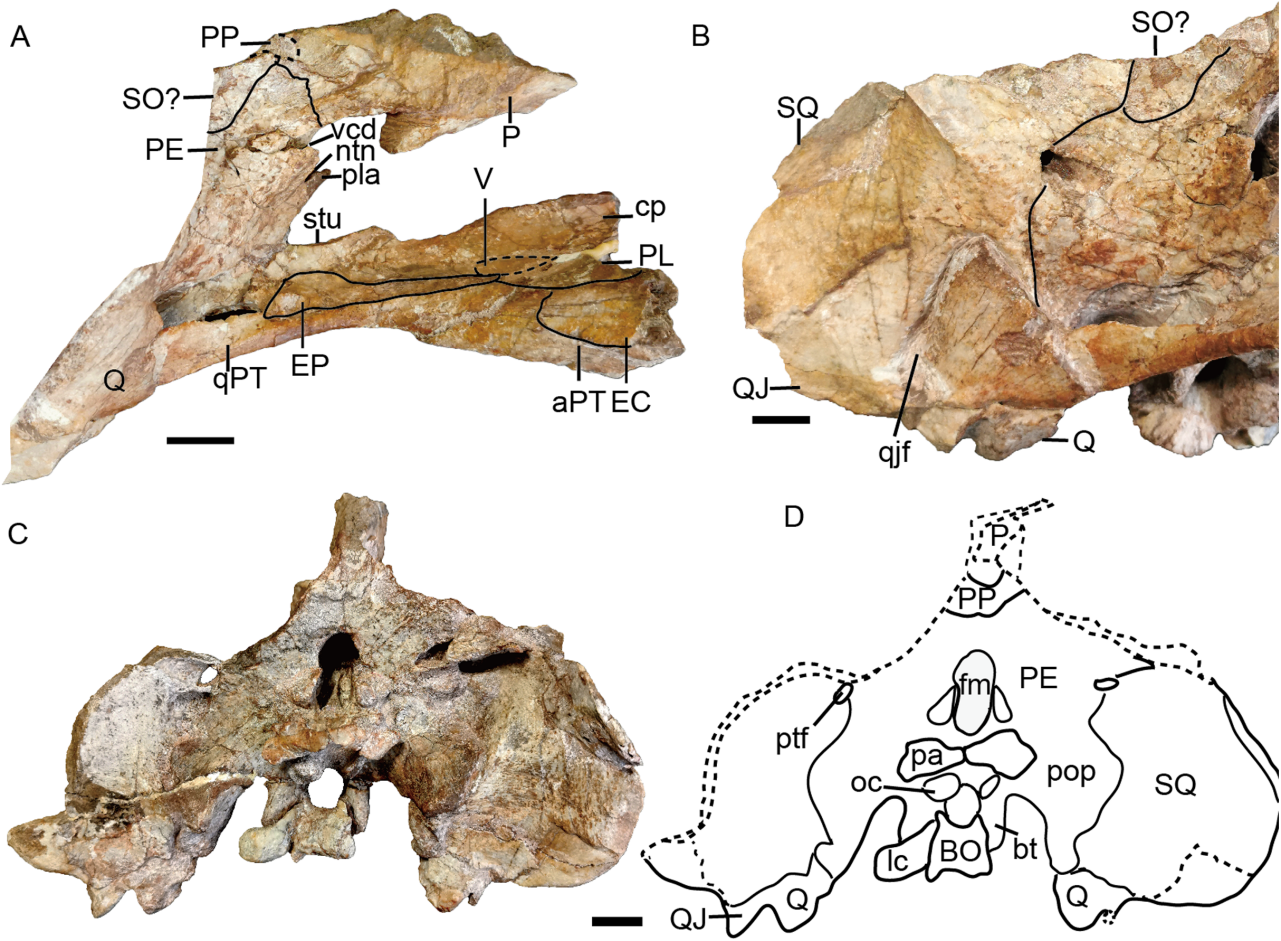
*Turfanodon jiufengensis* from the Naobaogou Formation, holotype (IVPP V 26038), skull. (A) posterior part in right lateral view; (B) right half of occiput in anterior view; (C) photo and (D) drawing of occiput in posterior view. Abbreviations: aPT, anterior ramus of the pterygoid; BO, basioccipital; bt, basal tuber; cp. cultriform process; EC, ectopterygoid; EP, epipterygoid; fm, foramen magnum; LC, lateral condyle; ntn, notch for trigeminal nerve; oc, occipital condyle; P, parietal; pa, proatlas; PE, periotic; PL, palatine; pla, pila antotica; pop, paroccipital process; PP, postparietal; ptf, post-temporal fenestra; Q, quadrate; QJ, quadratojugal; qjf, quadratojugal foramen; qPT, quadrate ramus of pterygoid; SO, supraoccipital; SQ, squamosal; stu, sella turcica; V, vomer; vcd, notch for the vena capitis dorsalis. Scale bars equal 2 cm. Photo/drawing credit: Jun Liu.

No complete squamosal is preserved in any specimen. A complete zygomatic ramus of the squamosal is preserved on the right side in IVPP V 26035 and V 23299 (Figs. 7 and 8); the quadrate ramus is preserved in the holotype and IVPP V 23299 (Figs. 8 and 14). The squamosal extends lateral to the jugal and terminates anteriorly in a sharp tip that covers the posterior end of the suborbital portion of the maxilla in lateral view (Fig. 6). The squamosal greatly expands in width posterior to the postorbital bar and becomes more vertically-oriented at the posterior end of the subtemporal zygoma, as in *Turfanodon bogdaensis*. The posterior contact between the zygomatic and quadrate squamosal rami forms a broadly-rounded arc, similar to the condition in *Daptocephalus* spp. and *T*. *bogdaensis*. The anterolateral surface of the quadrate ramus forms a fossa for the attachment of the M. adductor mandibulae externus lateralis (King, 1981a). Posteriorly, the squamosal contributes to the lateral edge of the occipital plate, making up roughly half of its transverse width (Fig. 14). The lateral edge of the squamosal attenuates in thickness laterally, and extends somewhat posterior to the middle portion of the occipital plate (Figs. 6 and 7). The squamosal surface is depressed lateral to the post-temporal fenestra. Anteroventrally, the squamosal forms a pocket for receiving the quadrate and quadratojugal.

The prefrontal occupies the anterodorsal corner of the orbit. It extends anteriorly to approach the level of the posterior margin of the external nares (Figs. 4 and 6). Within the anterior orbital wall, it extends medioventrally towards the maxilla in IVPP V 23879 (Figs. 4B and 4D), but not in IVPP V 26035 (Figs. 6 and 7). Posteriorly, it is separated from the postorbital by the frontal. The prefrontal boss, forming the anterodorsal rim of the orbit, is weakly developed but is clearly separate from the nasal boss (Figs. 3, 4 and 6). The surface of the prefrontal is also sculptured, and the foramina are fewer but larger than on the nasals of IVPP V 23879.

The frontals are only completely preserved in IVPPV 26035, but their anterior sutures are unclear in this specimen (Fig. 6). In other specimens, the frontals send sharp triangular medial processes to meet the premaxilla around the level of nasal bosses (Fig. 5). Laterally, the frontal forms a large portion of the dorsal orbital margin (Fig. 6). Posteriorly, the frontals constrict in width between the postorbitals and contact the parietals lateral to the pineal foramen and preparietal (Figs. 6 and 8). Ventromedially, the frontals contact the orbital plate (sphenoid) (Figs. 6 and 13). The orbital plate is formed by the fused orbitosphenoid and mesethmoid (Cluver, 1971), and it completely divides the orbits. Posteriorly, it has a notch as in *Lystrosaurus*, but unlike the condition in *Taoheodon* (Cluver, 1971; Liu, 2020). The notch is deep in the holotype but shallower in IVPP V 26035. The orbitosphenoid portion of the orbital plate is interpreted as bracing the mesethmoid portion ventrally (Fig. 13). The orbital plate is supported ventrally by the anterior process of the cultriform process of the parasphenoid and possibly the presphenoid.

A distinct postfrontal is absent, but it is possibly fused with the postorbital. The postorbital makes up the entire anterior and medial margin of the temporal fenestra, contributing to the postorbital bar and intertemporal bar. The postorbital bar is greatly twisted (dorsal surface continuous with medial surface) and expands in width ventrally to overlie the jugal, as in *Turfanodon bogdaensis* (Fig. 10D) and *Daptocephalus leoniceps* (Kammerer, 2019). The anterodorsal surface of the postorbital is tilted medially. The posterior edge of the medial (dorsal) side of the postorbital bar and the lateral surface of the intertemporal bar would have served as an attachment site for jaw adductor musculature. The intertemporal bar is narrow and forms a sharp parietal crest, whose lateral surface is more vertically-oriented than horizontally. On the ventral surface of the intertemporal bar, there is a small fossa formed by the ventral surface of the postorbital and lateral surface of the parietal. The postorbital extends posteriorly as a curved process along the posterior margin of the temporal fenestra, overlying the occiput.

Posterior to the frontal, the dorsal surface of the skull is completely eroded in the holotype but is preserved in other specimens (Figs. 3, 6 and 9). The single preparietal lies in a concave area anterior to the pineal foramen, surrounded by the frontals anteriorly and the parietal posteriorly. It looks like a ‘three-toe’ footprint, having one larger median process and two smaller lateral processes in IVPP V 26035 (Figs. 6 and 8). This bone exhibits additional small lateral processes on the anterior margin (making a zig-zag suture with the frontal) in IVPP V 23880 (Fig. 9). In this specimen the dorsal bone surface has been eroded off so that the suture is visible in section; the differing shape may be attributable to this being a deeper part of the suture.

The parietals are barely visible dorsally, with only a little exposure of short anterior processes around the pineal foramen (Fig. 6). In IVPP V 26035, due to erosion of the postorbital, part of the parietal is visible at the posterior end of the intertemporal bar. In *Turfanodon bogdaensis*, the parietals have narrow exposure between the postorbitals (Li, Cheng & Li, 2000). Laterally, the parietal is exposed beneath the postorbitals, and forms part of the dorsal portion of the braincase (Fig. 6). Ventrally, it contacts the ascending process of the epipterygoid from the lateral side. Posteriorly, it contacts the postparietal and periotic (Fig. 14).

The vomer is almost complete in the holotype and IVPP V 26035 (Figs. 7 and 12). Its anteriormost part lies behind the median palatal ridge of the premaxilla, where it suddenly narrows in width. Anteriorly, the vomer is a blade-like structure. On its dorsal side, the vomer extends far anteriorly as a ridge below the parasphenoid (Fig. 13). Posteriorly, it is divided as paired, vaulted laminae curving dorsolaterally to contact the palatines laterally and pterygoids posterolaterally. Medially, it surrounds an elongate, ‘teardrop’-shaped interpterygoid vacuity (narrow end anterior), bearing thin ridges along the margin of the vacuity.

The palatine is almost complete in three of the specimens (Figs. 7, 9 and 12). It extends anteriorly to the level of the tusks. It is separated from the premaxilla by a narrow medial process of the maxilla. The raised palatine pad does not appear to be especially rugose. The anteromedial corner of the bone is angled ventrally in IVPP V 23880 and on the left side of IVPP V 26035 (Figs. 7 and 9). The smooth, laminar posterior portion of the palatine forms part of the lateral wall of the choanae. The paired palatines are separated by the vomer. The lateral palatal foramen is not observed, possibly because of poor preservation of this area.

The right ectopterygoid is preserved in the holotype (Fig. 14), and the suture of the left ectopterygoid can be clearly traced in IVPP V 26035 (Figs. 6 and 7). It lies posterior to the maxilla, on the lateral surface of the pterygoid, and is close to but does not contribute to the labial fossa.

The large pterygoids are roughly X-shaped, composed of anterior and quadrate rami united by a median pterygoid plate (Figs. 7 and 12). The anterior pterygoid rami are bowed lateral to the palatines and form part of the external wall of the choanae. A narrow keel is restricted to the anterior tip of the anterior ramus in larger specimens, and the posterior part of the anterior ramus is rounded. However, in the small specimen IVPP V 23880, the anterior ramus is roughly equivalent in transverse width throughout its length (Fig. 9). The median pterygoid plate has an indistinct, thin crista oesophagea on the ventral surface. Paired ridges extends anteriorly from the crista oesophagea onto the anterior pterygoid rami (Fig. 7C). The quadrate rami are thin, ribbon-like structures extending from the median pterygoid plate toward the quadrates at a ~22° angle relative to the long axis of the skull. The quadrate ramus is widened distally and turns from dorsolaterally facing to ventrolaterally where it wedges between the quadrate and periotic. Dorsally, the pterygoid supports the parasphenoid which sends a median, laminar cultriform process forward, where it is wrapped by the palatine and underlain by the vomer (Fig. 6D).

The epipterygoid rests on top of the pterygoid. Only the anteroposteriorly elongated footplate is preserved in the holotype, and there is no dorsal process near the anterior tip of the footplate (Fig. 14A). The left epipterygoid is almost complete in IVPP V 26035 (Figs. 6C and 6D), preserving the long, thin ascending process that expands dorsally to contact the parietal.

The posterior palate and braincase are best preserved in the holotype, IVPP V 26038. On the palatal surface, the parabasisphenoid is exposed posterior to the median pterygoid plate (Fig. 12). Near its anterior margin are paired, ventrally-directed internal carotid canals. Posteriorly, the ventral surface bears paired ridges (with a shallow depression between them) that curve posterolaterally and expand to join the basal tubera. The parabasisphenoid bears an elongate cultriform process anteriorly, extending above the vomer and possibly reaching the premaxilla (Figs. 6, 13 and 14). On its dorsal surface, a longitudinal ridge divides the sella turcica, so there cannot be a single dorsal exit for the carotid canals as argued to be the case for all non-kannemeyeriiform dicynodonts by Surkov & Benton (2004). The basal tuber is anteroposteriorly elongate with relatively narrow edges and is angled somewhat ventrolaterally (Fig. 12). It is almost entirely formed by the basioccipital, except for the anterior edges, which are composed of parabasisphenoid. The intertuberal part of the basioccipital is damaged and the tri-radiate occipital condyle is incomplete, with the ventral portion formed by the basioccipital displaced and preserved between the basal tubera (Figs. 12 and 14).

The basioccipital, exoccipital, supraoccipital, opisthotic and prootic are fused to form a single periotic element (Figs. 7 and 14). This element forms the central part of the occiput. Its dorsal margin contacts the postparietal, tabular, and squamosal. The occiput is inclined anteriorly in the holotype but not in IVPP V 26035, likely due to compression during fossilization. The paroccipital process is a transversely short but dorsoventrally tall structure, with a strongly concave ventral edge and a broad depression on its dorsolateral posterior surface, below its contribution to the margin of the post-temporal fenestra. The post-temporal fenestra lies at the level of the center of the foramen magnum. The occiput is incomplete on the left side dorsal to the post-temporal fenestra in the holotype. The supraoccipital portion above the foramen magnum is weakly depressed on its posterior face. On the anterior surface of the periotic, there should be a notch for the vena capitis dorsalis near its border with the parietal based on the condition in related dicynodonts (Surkov & Benton, 2004). In the holotype and IVPP V 26305, only a wide, shallow notch is observed (Figs. 6 and 14). The notch for the trigeminal nerve is a small, V-shaped structure, bordered anteriorly by the distinct pila antotica. This area looks like a fenestra between the supraoccipital, parietal, and epipterygoid (Fig. 6).

The quadrate and quadratojugal are fused as a large complex anteroventral to the quadrate ramus of the squamosal (Figs. 12 and 14). Although the complex is incomplete on both sides in the holotype, they are complementary and together provide a nearly complete view of the morphology. This complex has the general morphology for dicynodonts (King, 1988). The quadratojugal forms a broad but thin plate, which is mostly occluded in posterior view by a ventral process of the squamosal, but partially exposed by damage to the latter bone. The quadratojugal is separated from the dorsal portion of the quadrate by a long, narrow quadratojugal foramen. The articular surface of the quadrate is made up of lateral and medial condyles separated by a trochlea ventrally. The quadrate is angled such that the medial condyle is situated somewhat posterior to the lateral one. The right lateral condyle is broken and pressed up against the basioccipital (Fig. 12). The dorsal process is roughly triangular in shape, with a nearly straight anterior edge that contributes to a sharp, short anterior process along the quadrate process of the pterygoid (Fig. 13B). If this is natural, it differs from other Permian dicynodonts, in which the dorsal edge of the quadrate is rounded.

The postparietal (or interparietal) lies posterior to the parietal and sends a pair of short anterior processes bounding the parietal laterally (Figs. 7 and 14). It is only partially preserved in the holotype but nearly complete in IVPP V 26035. In the occipital plate, it forms a concave area above the periotic.

The tabular lies lateral and ventral to the postparietal, dorsal to the supraoccipital portion of the periotic and medial to the squamosal (Fig. 7). It does not contribute to the post-temporal fenestra (Fig. 14).

### Mandible

**Figure 15:**
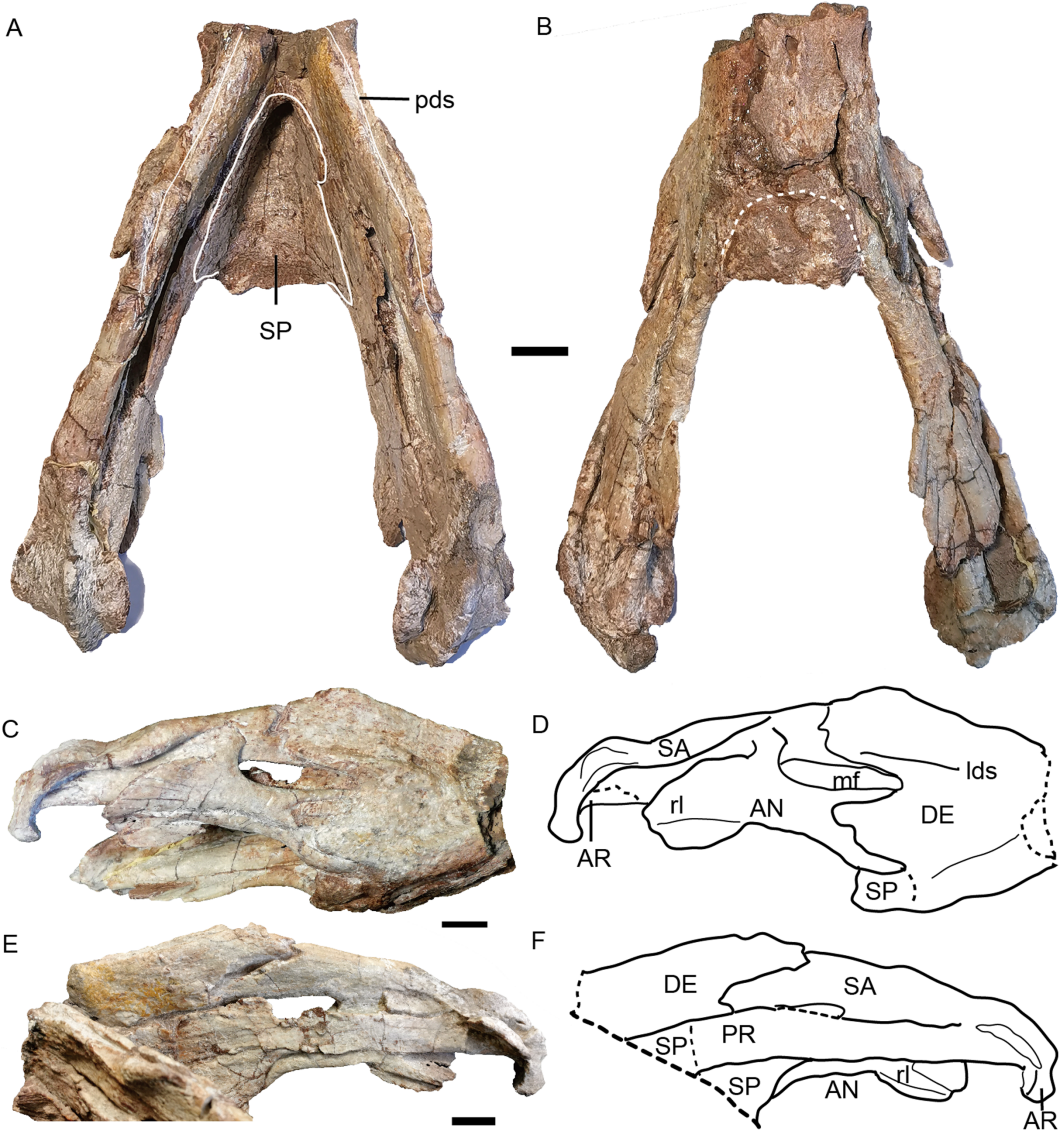
*Turfanodon jiufengensis* from the Naobaogou Formation, holotype (IVPP V 26038), lower jaw. Photos in (A) dorsal and (B) ventral views; (C) photo and (D) drawing of right ramus in ventrolateral view; (E) photo and (F) drawing of right ramus in dorsomedial view. Abbreviations: AN, angular; AR, articular; DE, dentary; lds, lateral dentary shelf; mf, mandibular fenestra; PR, prearticular; rl, reflected lamina of angular; SA, surangular; SP, splenial. Scale bars equal 2 cm. Photo/drawing credit: Jun Liu.

The mandibles are almost complete except for the dentary symphysis in the holotype (Fig. 15). The dentary is better preserved on the right side in IVPP V 23299, but the dorsal tip of the symphysis is still incomplete (Fig. 8). The mandible is edentulous as in most dicynodonts.

The dentary is a large, robust bone made up of the fusion of two rami. The dorsal edges on both sides of the symphysis are nearly parallel, but the two rami are divergent posterior to the symphysis (Figs. 8B and 15A). The lateral and anterior surface of the symphysis is rugose. The lateral surface of the symphysis corresponds to the inner surface of the caniniform process. The anterior and lateral faces of the dentary are clearly demarcated by sharp ridge in the holotype (Figs. 15C and 15D). Such a ridge is not observed in IVPP V 23299, possibly due to the lateral compression (Fig. 8). On the dorsal surface of each ramus, a midline groove, the posterior dentary sulcus, runs along the length of the dorsal margin (Fig. 15A). The dentary dorsal margin forms two sharp ridges along the groove. These would have been covered by keratin and used to cut food in life. On the lateral surface, the dentary is bifurcated into two processes above and below the mandibular fenestra (Figs. 8 and 15). Above the fenestra, the dentary has a longer posterior process, which slightly decreases in dorsoventral height posteriorly but still has a tall free edge at its contact with the surangular. The posterior margin of this process bears a very small, pointed posterior process near mid-height on its right lateral side, giving its posterior edge a ‘wavy’ appearance (Fig. 15). At the anterodorsal edge of the mandibular fenestra, there is lateral dentary shelf. It is dorsoventrally narrow but well-developed. It extends anterior to the mandibular fenestra for the same length as above the mandibular fenestra. Its anterodorsal edge is developed into a rounded swelling. Below the mandibular fenestra, the posterior process of the dentary is triangular and fits in a deep lateral facet on the angular. The mandibular fenestra is elongate, rounded at its anterior and posterior edges, and bordered dorsally by the dentary and surangular and ventrally by the angular in lateral view.

Like the dentary, the splenial is also a single fused element (Fig. 15A). It forms all of the dorsal surface and postero-ventral surface of the posterior (ventral) portion of the symphysisly (Figs. 15A and 15B). The splenial extends posteriorly along the medial face of the angular and the prearticular, terminating in a tapered process overlying the angular.

The angular extends anteriorly as a thin ventral process between the splenial and dentary and contributes to the symphysis (Figs. 15C and 15D). Dorsally, it comprises a flattened, strip-like element covered by dentary laterally and prearticular medially. It bears a posteriorly-directed reflected lamina below the posterior edge of the fenestra. The reflected lamina is large and rounded, with only a central groove bisecting its lateral face (Figs. 15C–15F). Its dorsal portion has a convex surface while the ventral portion is concave. The ventral margin of the lamina is directed posteroventrally. Medially to the reflected lamina, the angular extends further posteriorly to contact the articular.

The surangular forms the dorsal margin of the jaw posterior to the dentary (Figs. 15C–15F). It is well exposed above the mandibular fenestra, but only as a narrow strip posteriorly, above the angular in lateral view.

The prearticular is a long, ribbon-like element, which nearly completely covers the mandibular fenestra medially (Figs. 15E and 15F). Its anterior terminus is uncertain because it is covered by the splenial medially and the dentary laterally.

The articular consists of lateral and medial condyles around a median trochlea, where it would articulate with the quadrate. A short retroarticular process is only preserved on the right side in the holotype (Fig. 15).

### Axial skeleton

Most of the postcranial elements including the vertebrate column are exposed in dorsal view in the holotype (Fig. 1). The nearly complete vertebrate column is preserved on three blocks. On the first block, there are 13 presacral vertebrae; on the second block, there are 16 dorsal vertebrae; and on the last one, there are 6 sacral vertebrae and 15 caudal vertebrae. So, the preserved vertebral column is composed of 29 presacral vertebrae (6 cervicals and 23 dorsals), 6 sacrals, and 15 caudals, for a total vertebral count of 50, the greatest number currently known for a dicynodont (Table 2; although it should be noted that complete axial columns are known for very few taxa).

**Table 2:** Comparison of vertebral count among various anomodonts.

Taxa	T	Psac	Cer	Dor	Sac	Cau	References
*Suminia getmanovi*	>78	23	6	17	3	>52	Fröbisch & Reisz (2011)
*Patranomodon nyaphulii*			6		3		Rubidge & Hopson (1996)
*Diictodon feliceps*	45	27	6	21	4	14	Ray & Chinsamy (2003)
*Eosimops newtoni*	47	29	6	23	3	15	Angielczyk & Rubidge (2013)
*Robertia broomiana*		~26			3	>11	King (1981b)
*Endothiodon bathystoma*	>40	>25	>5	≥20	5	>10	Maharaj, Chinsamy & Smith (2019)
*Dicynodontoides nowacki*	>35	27	6	21	5	>3	Cox (1959)
*Dicynodontoides recurvidens*					4		Griffin & Angielczyk (2019)
*Cistecephalus microrhinus*	>33	30	5	25	3		Cluver (1978)
*Pristerodon mackayi*	>31	26	6	20	5		Watson (1960)
*Niassodon mfumukasi*				>10	4	>3	Castanhinha et al. (2013)
‘*Aulacocephalodon peavoti*’	>36	26			5	>5	Olson & Byrne (1938)
*Oudenodon bainii*	>44	29	6	23	5	>10	SAM-PK-K-5477
‘*Dicynodn trigonocephalus*’	45	26	6	20	5	14	King (1981a)
*Peramodon amalitzkii*	>41	30	6	24	6a		Sushkin (1926)
*Turfanodon jiufengensis*	50	29	6	23	6	15	This paper
*Lystrosaurus murrayi*	42	26	6	20	6	10	Ray (2006)
*Lystrosaurus hedini*	>37	26	6	20	5	>6	Young (1935)
*Shansiodon wangi*	>40	~30	>3	>17	5		Yeh (1959)
*Tetragonias njulilus*		>19			5b		Cruickshank (1967)
*Sinokannemeyeria yingchiaoensis*			7	>9	5		Sun (1963)
*Parakannemeyeria youngi*	>44		>5	20	5	14	Sun (1963)
*Kannemeyeria simocephalus*	~46	25			6	15	Cruickshank (1975)
*Wadiasaurus indicus*	>30	24/25	6/7	18	5c		Bandyopadhyay (1988)
*Dinodontosaurus turpior*	43-44	24	5	19	6d	~15	Cox (1965)
*Sangusaurus parringtonii*					7		Angielczyk, Hancox & Nabavizadeh (2017)
*Placerias gigas*	~45		7	~25	7	~6	Camp & Welles (1956)

**Notes:**

The current taxonomic status of *Aulacocephalodon peavoti* and *Dicynodn trigonocephalus* are uncertain. Some sacral numbers are revised according to Griffin & Angielczyk (2019), the original numbers are: a, 4; b, ?; c, 5/6; d, 5.

T, total; Psac, presacral; Cer, cervical; Dor, dorsal; Sac, sacral; Cau, caudal.

**Figure 16:**
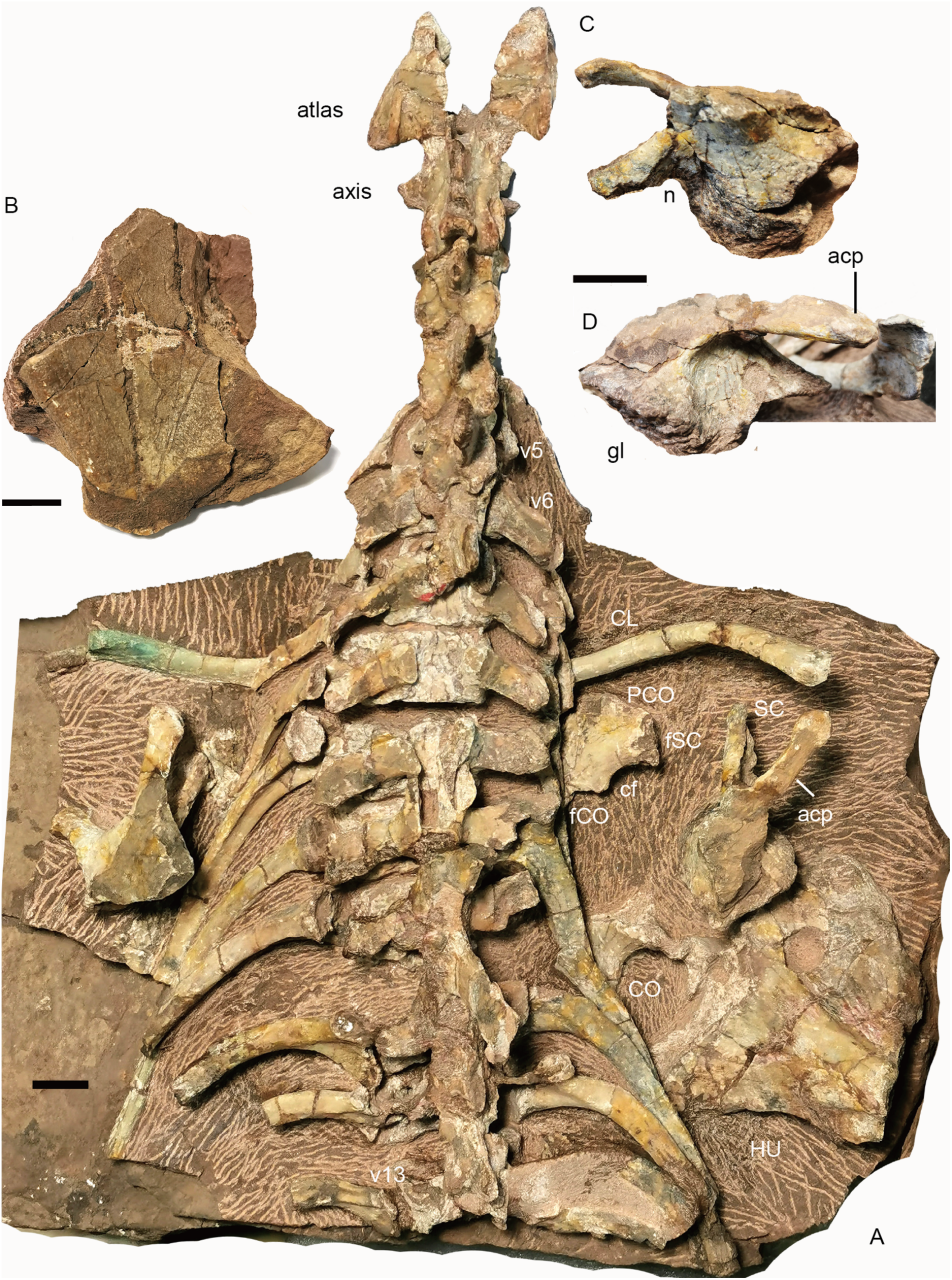
*Turfanodon jiufengensis* from the Naobaogou Formation, holotype (IVPP V 26038). (A) cervical and anterior dorsal vertebrae, ribs, and pectoral girdles in dorsal view; right scapula: (B) dorsal end in lateral view; base in (C) dorsomedial and (D) lateral views. Abbreviations: acp, acromion process; cf, coracoid foramen; CL, clavicle; CO, coracoid; fCO, facet for coracoid; fSC, facet for scapula; gl, glenoid; HU, humerus; n, notch; PCO, procoracoid; SC, scapula; v, vertebra. Scale bars equal 2cm. Photo credit: Jun Liu.

**Cervical region.** The 6th vertebra is assumed to be the last cervical because it still lies anterior to the level of the clavicle and bears short ribs (Fig. 16). The cervical number generally is six in anomodonts (Table 2), although five have been reported in *Cistecephalus* and *Dinodontosaurus* (Cluver, 1978; Cox, 1965) and seven were reported in the Triassic kannemeyeriiforms *Wadiasaurus*, *Sinokannemeyeria*, and *Ischigualastia* (Bandyopadhyay, 1988; Camp & Welles, 1956; Sun, 1963). The centra are not preserved from the atlas to the third cervical.

**Figure 17:**
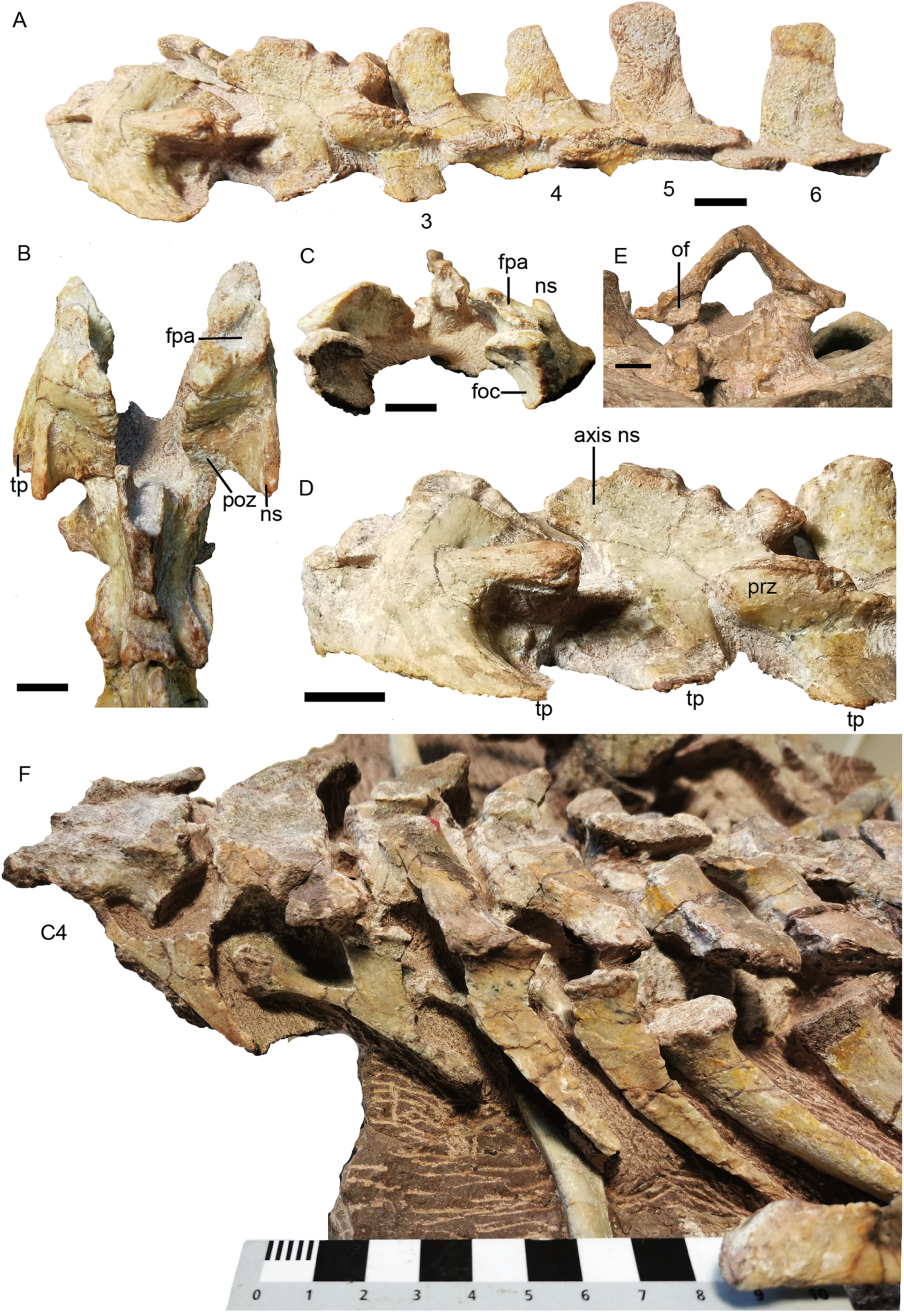
*Turfanodon jiufengensis* from the Naobaogou Formation, holotype (IVPP V 26038). (A) Incomplete cervical vertebrae in lateral view; atlas-axis complex in (B) dorsal, (C) anterior, and (D) lateral views; (E) proatlas in anterior view; (F) cervical and anterior dorsal ribs in left lateral view. Abbreviations: foc, facet for occipital condyle; fpa, facet on the atlas neural arch for the proatlas; ns, neural spine; of, proatlas facet to contact the occiput; poz, postzygapophysis; prz, prezygapophysis; tp, transverse process. Scale bars equal 1 cm. Photo credit: Jun Liu.

The atlas–axis complex preserves the paired proatlases, the paired atlantal neural arches, and a fused axial neural arch, with the intercentra and pleurocentra missing (Fig. 17). The cervical neural arches are all similar in anteroposterior length as measured from the prezygapophyses to the postzygapophyses (~46 mm).

The proatlases are well preserved at the back of the holotype skull, and are fused dorsally along the midline (Fig. 17E). Each element is thickened ventrally, and its anterior surface bears a facet contacting a facet lateral to the foramen magnum on the occiput. The atlantal neural arches are a pair of separated elements with their postzygapophyses articulating with the axis (Fig. 17). Anteriorly, the proatlas facet lies on the dorsal surface. Ventromedially, a tongue-shaped facet is present to receive the dorsolateral surface of the occipital condyle (Fig.17C). Its median side extends anteroposteriorly as a lamina and covers the neural canal. Posteriorly, the neural arch flares out and becomes more robust to form the postzygapophysis (Figs. 17B and 17D). The neural arch curves laterally to give rise to a prominent transverse process that projects posterolaterally. Its distal end bears no rib facets. The atlantal neural spine lies lateral to the postzygapophysis and is much lower than the other neural spines (Figs. 17A and 17B). The axis features an anteroposteriorly expanded neural spine, which is drawn up into rugose bosses anteriorly and posteriorly. Its prezygapophysis is a flat plate covered by the atlantal postzygapophysis. The postzygapophysis is well developed, with its articular surface facing ventrolaterally (Fig. 17D). The transverse process is located rather low and is poorly preserved.

The third cervical also does not preserve its centrum, and its transverse processes are directed slightly ventrally and posterolaterally (Fig. 17A). Its neural spine is anteriorly inclined, increasing in length upwards and forming a rugose, convex tip. From the 4th to 6th cervicals, the centrum does not show differentiation in its anteroposterior length (~24 mm), the transverse processes are directed posterolaterally, and the zygapophyses are well developed (Figs. 16 and 17). The neural spines decrease in anteroposterior dimensions dorsally, especially on the 4th cervical which has a narrow tip. The heights of the neural spines increase posteriorly, and the neural spines on the 5th and 6th cervicals are more robust. The neural spine is anteriorly inclined on the 4th and 5th cervicals, but nearly upright on the 6th cervical.

The 4th to 6th cervical ribs are better exposed on the left side of the skeleton (Fig. 17F). Their lengths increase posteriorly (2.5, 3.5, 4.5 cm). The well-preserved ribs are dichocephalous with a clear distinction between the capitulum and tuberculum, and the shaft is slightly curved medially.

**Figure 18:**
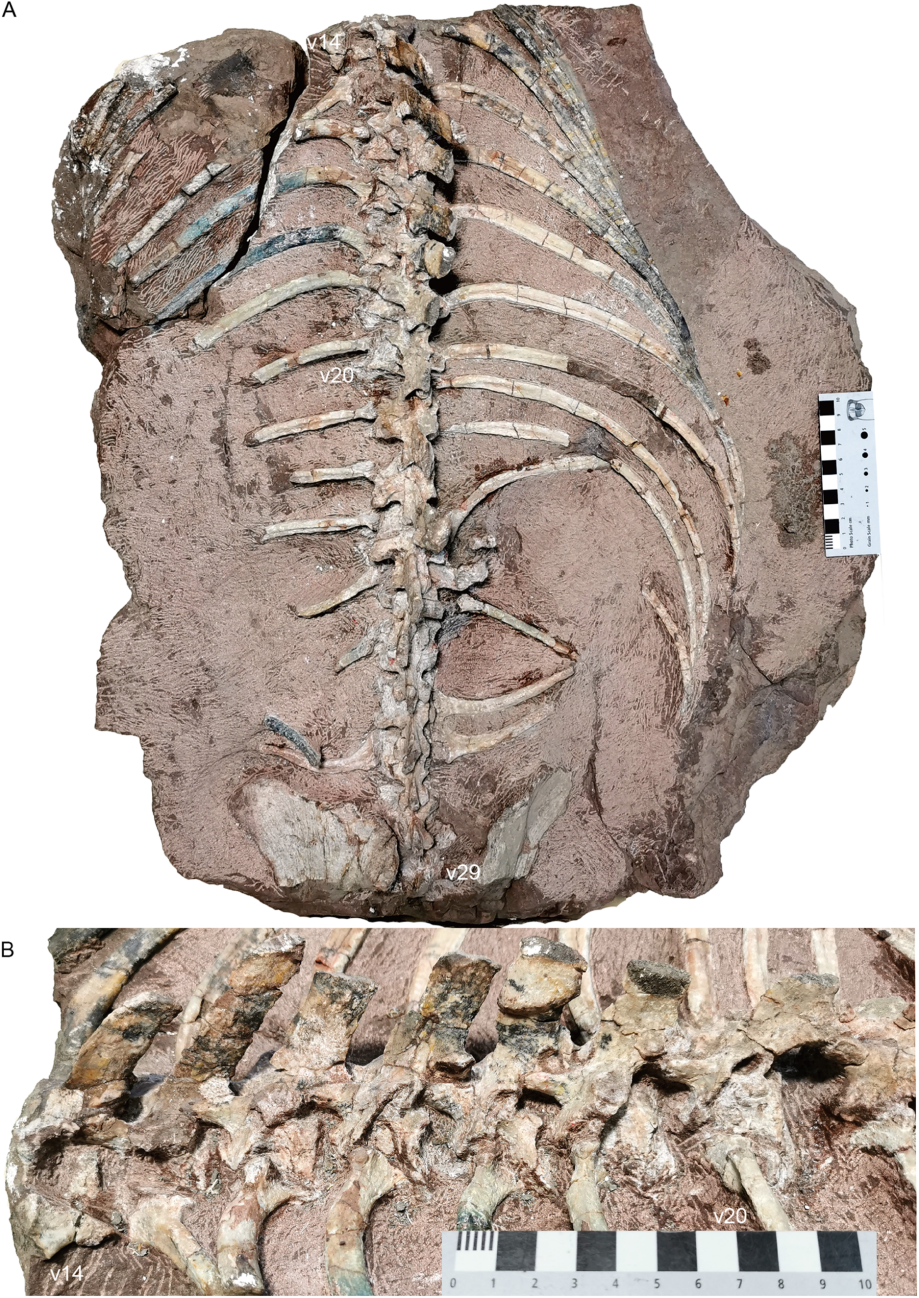
*Turfanodon jiufengensis* from the Naobaogou Formation, holotype (IVPP V 26038). Posterior dorsal vertebrae and associated ribs in (A) dorsal and (B) left dorsolateral views. Photo credit: Jun Liu.

**Dorsal region.** The 7th vertebra bears posterolaterally directed transverse processes, like the preceding cervicals but unlike the subsequent dorsal. Its ribs are much shorter (7.5 cm) than the more posterior ribs. From the 7th to 13th vertebrae, the neural arches are preserved incompletely (except for the 10th vertebra), with only the transverse processes being preserved in the 7th and 8th. The dorsals on the second block are nearly complete except the dorsal tip (Fig. 18).

The centra of the first and second dorsals are exposed in dorsal view, and the width of the centrum is greater than the length (Fig. 16). The transverse processes are directed posterolaterally on the 7th vertebra, but almost straight laterally starting at the 9th vertebra. The transverse processes are of consistent anteroposterior width for their entire length on the 7th vertebra, but they get wider towards their lateral tips on the 8th–10th. Their width decreases posteriorly, at least on the anterior dorsals. The transverse processes are poorly preserved on the middle dorsals. Starting at the 17th vertebra, the transverse processes are angled anterodorsally (Fig. 18). More posteriorly, they gradually extend ventrally on the centrum, face more dorsally, and merge with the anterior surface of the neural arch. Also, the rib facet is on the ventrolateral surface of the transverse process and the anterior border of the lateral face of the centrum. On the 20th vertebra, the transverse processes are wide in dorsal view, with a free, thin dorsolateral end that is directed posterodorsally. Starting at the 21st vertebra, the transverse processes extend posteriorly beyond the posterior margin of the centra.

Compared to those on the cervicals, the zygapophyses on the middle dorsals are reduced and are more vertically orientated (Fig. 18B). The prezygapophyses also slightly cover the postzygapophyses laterally. This indicates both lateral and vertical movements were restricted. Starting at the 19th vertebra, the postzygapophyses are more horizontally oriented and wider, and extend far behind respective centra. Starting at the 22nd vertebra, the postzygapophyses are long and broad and form a nearly horizontal plate lateral to the base of the neural spine. These imply a reduction in the lateral flexibility of the anterior part of the thorax, and greater lateral flexibility in the abdominal region, similar to the condition in *Dicynodontoides*, *Diictodon*, and *Eosimops* (Angielczyk & Rubidge, 2013; Cox, 1959; Ray & Chinsamy, 2003).

All preserved dorsal neural spines are directed posterodorsally (Figs. 16 and 18). The only complete one (on the 15th vertebra) measures 6 cm in height. The neural spines are most robust in the middle section of the dorsal series. The neural spines on the 16th to 22nd vertebrae are considerably more anteroposteriorly expanded than those on the 14th and 15th vertebrae or on the posterior dorsals. Based on what is preserved, they do not appear to have tapered much dorsally.

The dorsal ribs are relatively well-preserved (Figs. 16–18). On the anterior dorsals, the capitula and tubercula gradually merge and become less distinguishable. The ribs of the 7th to 10th vertebrae are dichocephalous while others are holocephalous as in *Dicynodontoides* (Cox, 1958). The ribs are fairly robust starting at the 8th vertebra: the incomplete rib of the 9th vertebra measures 16 cm, and the complete exposed ribs measure ~30 cm. Although incomplete, the posterior dorsal ribs become slender and decrease in length.

**Figure 19:**
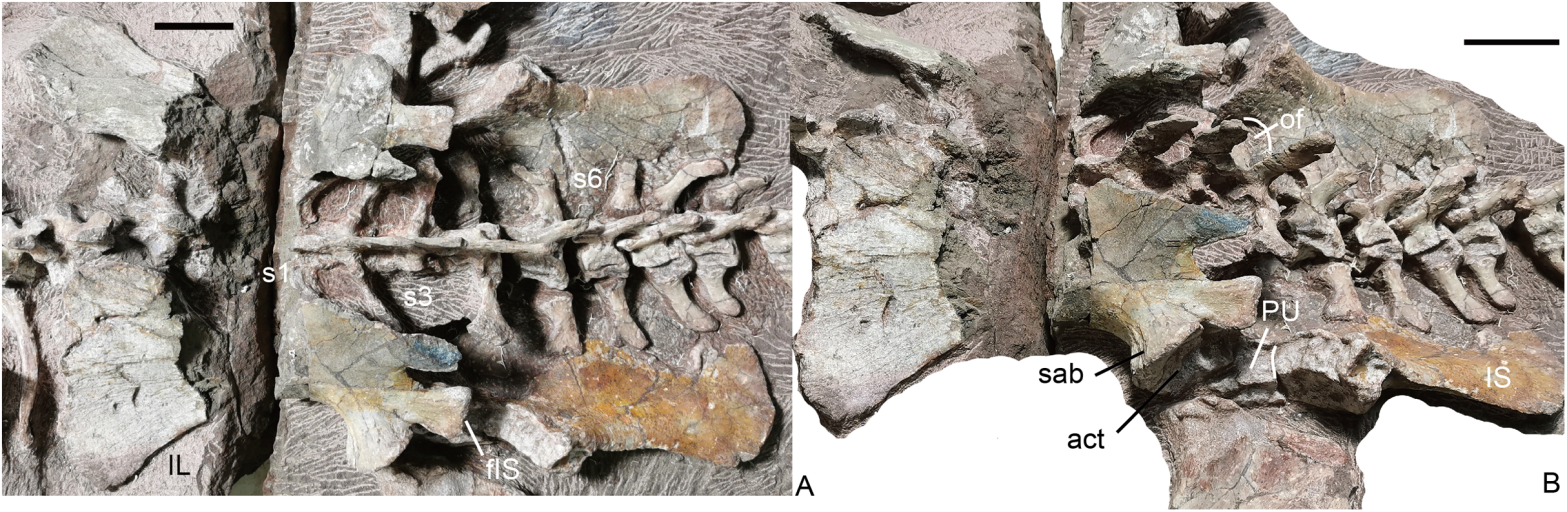
*Turfanodon jiufengensis* from the Naobaogou Formation, holotype (IVPP V 26038). Sacral vertebrae and ribs, and pelvis in (A) dorsal and (B) left dorsolateral views. Abbreviations: act, acetabulum; fIS, facet for ischium; IL, iliumm; IS, ischium; of, obturator foramen; PU, pubis; S, sacral; sab, supraacetabular buttress. Scale bars equal 4 cm. Photo credit: Jun Liu.

**Figure 20:**
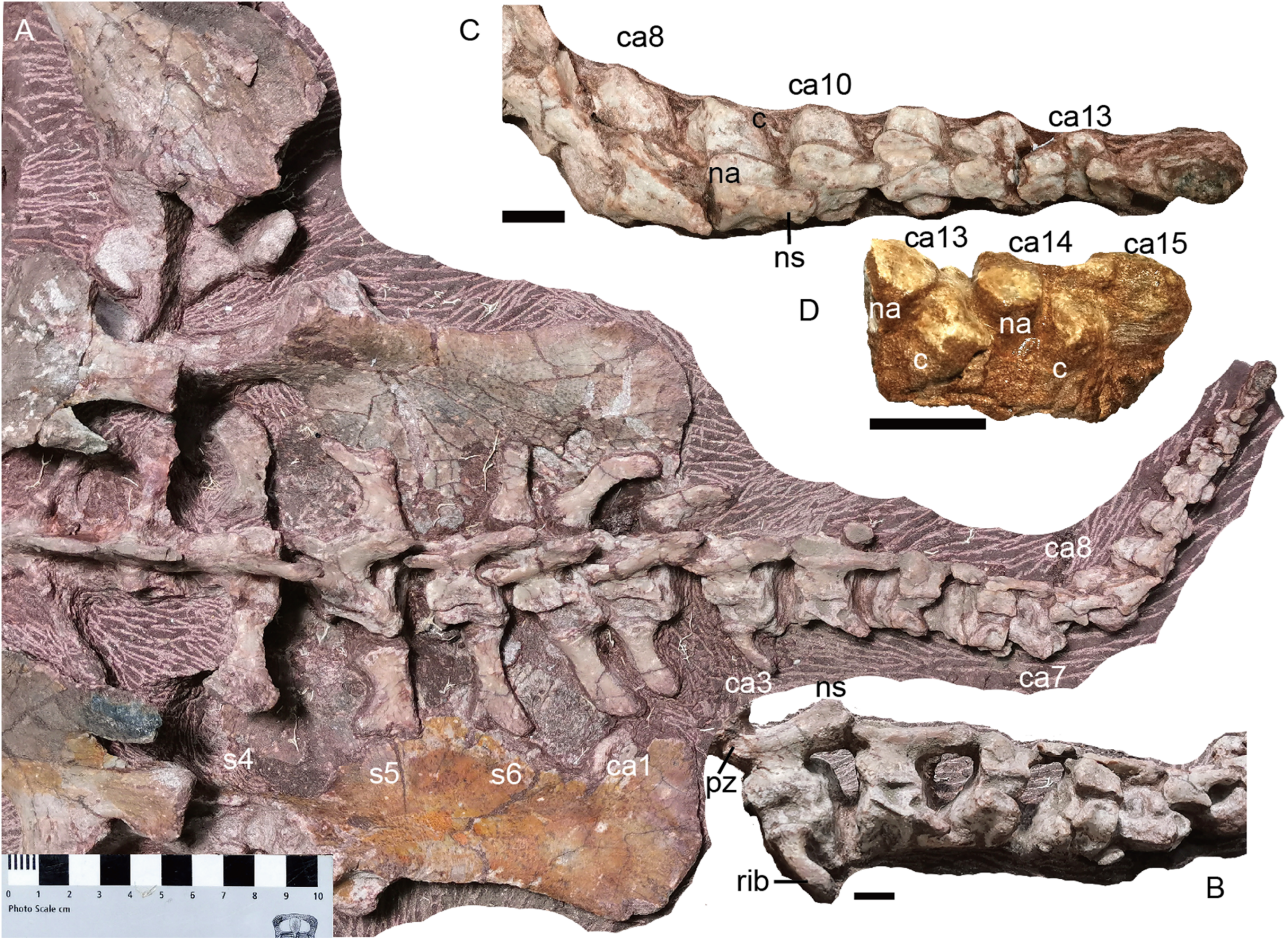
*Turfanodon jiufengensis* from the Naobaogou Formation, holotype (IVPP V 26038). (A) Complete tail mainly in dorsal view; (B) third to 7th caudals in left lateral view; (C) 8th to 15th caudals in dorsal view; (D) last three caudals in left lateral view. Abbreviations: c, centrum; ca, caudal; na, neural arch; ns, neural spine; pz, prezygapophysis; s, sacral. Scale bars equal 1 cm. Photo credit: Jun Liu.

**Sacral region.** Four sacral vertebrae are fused, although the first one is incomplete (Figs. 19 and 20). Based on the length of the ilium and the preserved sacral vertebrae, there should be 6 sacral vertebrae. It is assumed that there is no missing vertebra between the two blocks and the last sacral is the 35th vertebra.

In the sacral region, both the anterior and posterior zygapophyses are considerably reduced. The preserved zygapophyses are vertically orientated and extremely narrow transversely. The width of the second pair of prezygapophyses is much wider than that of the posterior pairs. Posteriorly, the neural spines of the sacral vertebrae decrease in anteroposterior length, and incline more posteriorly. The last two sacrals are distinctly smaller than the anterior four sacrals.

The sacral ribs are all expanded, but decrease in width posteriorly. The anterior four pairs are fused to the rib facets of the centrum, but the 5th and 6th pairs of ribs are free. The connections between the sacral ribs and the ilium were not fused. The lack of fusion between these structures may indicate immaturity at the time of death of the animal.

**Caudal region.** The complete tail, comprising of 15 caudal vertebrae, measures ~30 cm in length. It is mostly exposed in dorsal view (Fig. 20). The vertebrae gradually diminish in size towards the tip of the tail, including the centra, neural arch and neural spines. All adjacent centra are separated by a gap, indicating the presence of unossified intervertebral cartilage in life. In the last few caudals, the neural arch is separated from the centrum.

The zygapophyses are vertically orientated. The prezygapophyses are much wider than those of sacrals for the first and second caudals, then their proportional width and length decreases posteriorly along with their diminishing in absolute size. The prezygapophyses become indistinct from the 7th caudal on. The postzygapophyses migrate posteriorly along the ventral side of the neural spine, being positioned near the posterior end from the fourth caudal onwards.

The caudal neural spines are strongly posteriorly inclined. Other than the anterior three caudals, in which they are still slightly directed dorsally, all other neural spines are nearly horizontal. The neural spine extends posteriorly far beyond the posterior margin of its centrum. A neural spine is present on the 10th caudal, uncertain on the 11th and 12th caudals due to poor preservation, and absent on the 13th caudal.

The anteriormost three caudal vertebrae clearly bear ribs, and a piece of bone on the posterolateral corner of the 4th caudal centrum may represent a displaced rib. No rib is observed from the 5th caudal on. The caudal ribs are slender and slightly curved posteriorly. The first pairs of caudal ribs have a proximodistal length of ~4 cm. The ribs rapidly diminish in size posteriorly. No haemal arches can be observed.

### Limb girdles

**Pectoral girdle.** Preserved pectoral girdle elements include the paired clavicles and scapulae and the right coracoid plate (Fig. 16). Both scapulae are preserved in situ but are highly incomplete. They preserve the basal portion including the acromion process, but the scapular blade is only partially preserved on the right side (Fig. 16). The long acromion process is directed anteriorly and slightly laterally. The base of the scapula has a rounded glenoid facet for the humerus. Anteriorly, it extends slightly dorsally as a thin flange, whose ventral surface articulates to the procoracoid. On the medial side, a notch lies on its ventral margin, forming the scapular portion of the coracoid foramen (Fig. 16C). The scapular blade flares broadly dorsally.

The right coracoid plate is partially exposed its medial side, and the procoracoid is separated from the coracoid (Fig. 16A). Anteriorly, the procoracoid has a convex edge which is damaged. Dorsally, it has a facet for articulation to the scapula. Posterodorsally, it is notched by the large coracoid foramen. Its posterior margin is the place for articulation to the coracoid. The coracoid is only exposed at its posterodorsal corner. It is thickened dorsally.

The dorsal end of the left clavicle is damaged, while the right one is complete and approaches the acromion process (Fig. 16A). The distal end is expanded dorsally into a fan-shaped plate. The ventral portion of the clavicle is directed medially and slightly posteriorly.

**Pelvic girdle.** Both left and right pelves are preserved (Fig. 19). The ilia are split into two parts by the crack between the blocks, and the middle portion is eroded. The pubes are only partially exposed, but the ischia are well exposed in medial view. The ilia disarticulated from the sacrum after death, and have slipped slightly anteriorly relative to their original position. The puboischiadic plates were dorsoventrally flattened, which caused them to be detached from the ilia, and they are in contact with the ventral surfaces of the sacral and anterior caudal vertebrae and ribs. The following description is mostly based on the better-preserved left innominate.

The iliac blade is wide, subtriangular and concave laterally, similar to *Wadiasaurus* (Ray, 2006). The complete ilium measures ~18 cm in anteroposterior length. Although incomplete, it is observed that the dorsal edge of the iliac blade is formed by two slightly convex portions, and they intersect in an angle of ~100°. There is no notch on the preserved portion of the dorsal edge. The iliac blade has a long, tall preacetabular process. The postacetabular iliac process is short with a sharp caudal end. The ilium has a short, constricted neck above the acetabulum. It is ‘teardrop’-shaped in cross-section, with the anterior side wider than the posterior side. The neck is laterally expanded to form a supraacetabular buttress above and anterior to the acetabulum. Posteriorly, the buttress is separated from the posterior acetabular margin by a shallow supraacetabular notch. The anteroventral surface of the ilium is still articulated with the pubis, but the ischium has become disarticulated from the posteroventral surface of the ilium. The lack of fusion of the pelvic elements raises the possibility that this individual may still be a sub-adult despite its large size. The articular surface for the ischium is gradually expanded in width outwards and forms a distinct triangular tubercle laterally. The medial iliac surface is not exposed.

The ventral portion of the acetabulum is formed by the pubis and the ischium. The dorsal surface of the pubis articulates with the ilium and its lateral surface contributes to the acetabulum. The posterior side of the pubis articulates with the anterior side of the ischium. The obturator foramen lies between two bones, and only its dorsal margin is exposed in medial view.

The ischium has a posteriorly expanded dorsal surface for articulating with the ilium. At its posterodorsal corner, the edge of the ischium becomes markedly thicker and forms a roughly square facet which is strongly concave. Its medial side extends posteriorly as a sharp keel on the posterodorsal margin of the ischium. Laterally, the ischium forms the posterior part of the ventral portion of the acetabulum. Moving ventrally and posteriorly, the ischium rapidly flattens to form a fan-shaped flat plate. A bony connection between the two ischia appears to be present anteriorly, but the bones are separated posteriorly.

### Limbs

**Figure 21:**
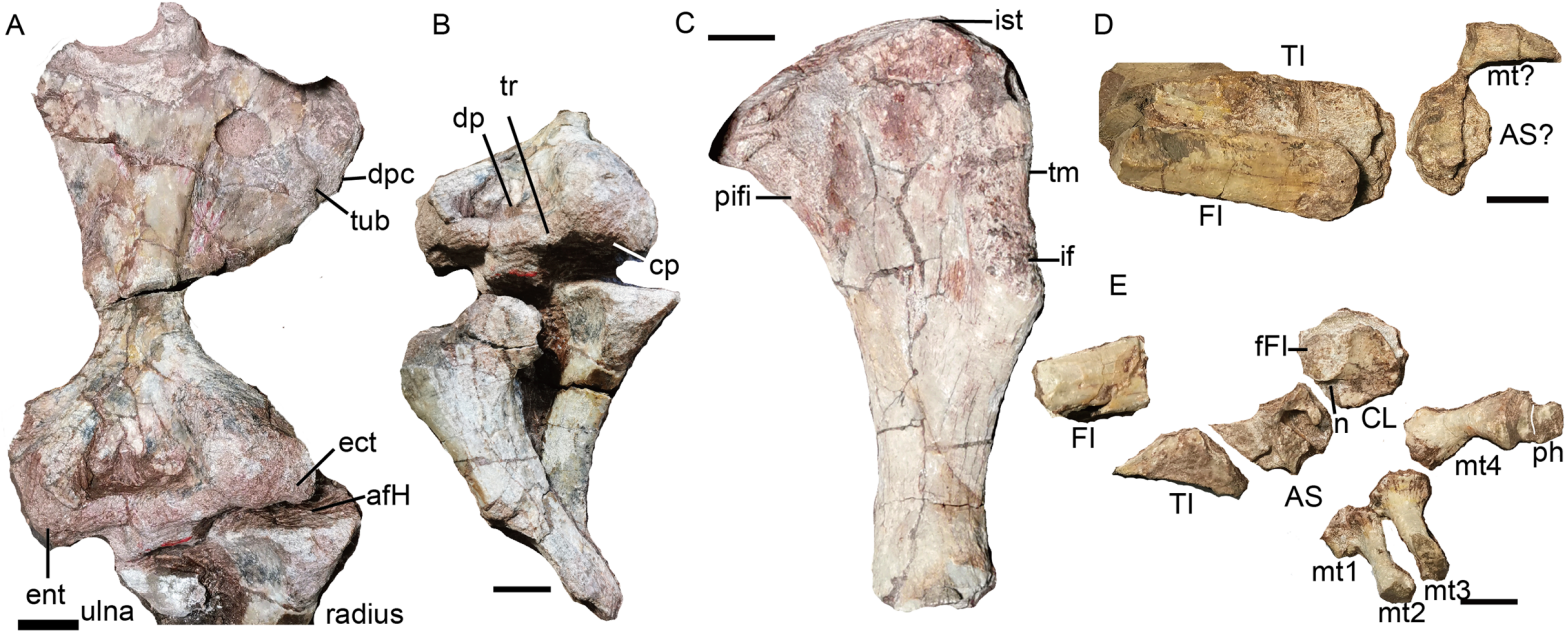
*Turfanodon jiufengensis* from the Naobaogou Formation, holotype (IVPP V 26038). (A) Right humerus in dorsal view; (B) humerus in dorsodistal view, ulna in lateral view, and radius in posterior view; (C) left femur in dorsal view; (D) right tibia, fibula and pes; (E) left tibia, fibula and pes in dorsal view. Abbreviations: afH, articular facet for humerus; AS, astragalus; CL, calcanem; cp, capitellum; dp, depression; dpc, deltopectoral crest; ect, ectepicondyle; ent, entepicondyle; fFI, facet for fibula; FI, fibula; if, M. ilio-femoralis; ist, M. ischio-trochantericus; mt, metatarsal; n, notch; ph, phalange; pifi, M. pubo-ischio-femoralis internus; TI, tibia; tr, trochlea; tub, tuberosity. Scale bars equal 2 cm. Photo credit: Jun Liu.

**Forelimb.** Only the incomplete right forelimb is preserved, comprising the humerus, ulna and radius (Fig. 21). The right humerus is mainly exposed dorsally. The proximal end is damaged and the articular surface is not preserved. The proximal portion is strongly dorsoventrally crushed. Although this has likely exaggerated the width of this portion somewhat, it was unlikely to be narrower than the distal portion when intact. The deltopectoral crest is massive and flared. On its anterior surface, a rugose tuberosity for M. deltoideus develops close to the ventral margin. The shaft is narrow and short. The entepicondyle extends further distally than the ectepicondyle. On the dorsal surface, a triangular median depression lies between two condyles, the area for the origin of M. triceps humeralis medialis (Angielczyk & Rubidge, 2013). The trochlea was damaged during preparation, and it seems extend to the dorsal surface. The capitellum is exposed in distal view.

The incomplete ulna (>12 cm) lost the olecranon process and the distal end. Its anterior surface in front of the sigmoid notch has a facet which receives the radius. The right radius could be complete, but is not fully exposed. It is longer than 10 cm. The proximal end is wider than the shaft. The proximal end has a concave facet for the capitellum (radial condyle) of the humerus.

**Hindlimb.** The proximal half of the left femur is complete, while the right femur is poorly preserved (Figs. 2 and 21). The description of this element is based on the left femur (Fig. 20C). The head of the femur is a hemispherical swelling, convex anteroposteriorly and flattened mediolaterally. It is offset on the long axis of the bone, and its articular surface faces dorsomedially. A muscle crest on the cranial edge, just distal to the femoral head, forms the site of insertion of M. pubo-ischio-femoralis internus. Lateral to the head, M. ischio-trochantericus inserted on a raised triangular area on the proximal femoral surface. The trochanteric major has a thick edge for the insertion site of M. ilio-femoralis on both anterior and posterior surfaces.

The tibia and fibula can be differentiated by their width, which is greater in the tibia (Ray, 2006). The right tibia is better preserved than the left one (Figs. 20D and 20E), but it is also partially covered by the right fibula. The distal half of the right fibula is preserved intact, whereas only the distal end of the left is present.

The only pedal elements from the right side of the body are a poorly preserved astragalus (?) and a partial metatarsal (Fig. 20D). The left pes preserves the astragalus, calcaneum, four metatarsals, and two phalanges of the same digit (Fig. 20E). The astragalus is broken. The calcaneum is a semi-circular bone. A rough, slightly convex surface on the proximal side forms the articular facet for the fibula. The posteromedial portion exhibits two protuberances separated by a notch; both correspond to the articular surfaces of the astragalus. The metatarsals may be 1–4 based on their position. The metatarsal 1 only preserves the proximal end, whereas metatarsal 4 is complete. The metatarsals are hourglass-shaped and the proximal end is wider than the distal end. They are slender, as in small and medium-sized dicynodonts such as *Diictodon feliceps*, *Eosimops newtoni*, and *Lystrosaurus georgi* (Angielczyk & Rubidge, 2013; Ray & Chinsamy, 2003; Surkov, Kalandadze & Benton, 2005), but different from the stockier metatarsals of larger dicynodonts such as *Wadiasaurus indicus* and *Parakannemeyeria youngi* (Bandyopadhyay, 1988; Sun, 1963). The preserved two phalanges are wide and short.

## Discussion

The most striking feature of these new specimens is elongate ascending process of the premaxilla that contacts to the anterior process of the frontal. This feature was previously only known in *Dinanomodon gilli* and *Turfanodon bogdaensis* among Permian dicynodonts. These specimens clearly differ from *D. gilli* but are similar to *T. bogdaensis* by having the premaxillary anterior tip squared off and the snout all and very steeply sloping.

Two specimens have been referred to *T. bogdaensis* other than the holotype IVPP V 3241 (Kammerer, Angielczyk & Fröbisch, 2011). The holotype of ‘*Dicynodon sunanensis*’ (IGCAGS V296) seems to differ slightly from IVPP V 3241 in the absence of a ridge below the naris on the lateral surface of the maxilla (Fig. 10). The holotype of ‘*Striodon magnus*’ (IVPP V 4694) is more poorly preserved, complicating identification of the diagnostic characters of *T. bogdaensis*. In the table below, all referred specimens of two species are compared for skull size and some cranial features (Table 1).

These specimens can be clearly divided into two morphologically distinct groups: *Turfanodon bogdaensis* (including IVPP V 3241 and IGCAGS 296), and *T. jiufengensis* (IVPP V 23299, 23879, 23880, 26035 and 26038). The former has an internarial region with a smooth dorsal surface, the dorsal tip of the premaxilla forming a pit on the dorsal surface, the lacrimal extending anteriorly beyond the prefrontal but not touching the septomaxilla, and parietals exposed in midline groove on the skull roof. The latter has an internarial region with a concave dorsal surface, a distinct premaxillary median ridge and raised nasal bosses, the dorsal tip of the premaxilla forming a low but convex surface, a lacrimal with anterior extension posterior to the prefrontal that contacts the septomaxilla, and a preparietal with anterolateral processes, and a narrow, crest-like median exposure of the parietal on the skull roof.

The median premaxillary ridge is present in the smallest specimen of *T. jiufengensis*, but its presence is uncertain in two medium-sized specimens because of poor preservation. The distinct ridge below the naris on lateral surface of maxilla is only observed in IVPP V 3241, and could be intraspecific variation (as it is absent in the otherwise comparable IGCAGS 296). The jugal has narrow exposure in lateral view only in the holotype of *T. jiufengensis*; more speciemns are needed to evaluate variation in this feature.

The largest specimen under consideration, IVPP V 4694 (‘*Striodon magnus*’), differs from all other specimens in having a low, wide occiput and rounded foramen magnum. Differences in occipital shape between this specimen and other skulls of *Turfanodon* was explained as taphonomic deformation by Kammerer, Angielczyk & Fröbisch (2011). Based on current evidence, it is parsimonious to tentatively refer IVPP V 4694 into *Turfanodon bogdaensis*, noting that this difference could also be due to ontogenetic change given the large size gap between IVPP V 4694 and the other specimens.

### Phylogenetic analysis

The phylogenetic position of *Turfanodon* has varied in different cladistic trees in recent years. *Turfanodon* was first proposed as the sister-taxon of Kannemeyeriiformes (Kammerer, Angielczyk & Fröbisch, 2011), then somewhere outside the clade of *Lystrosaurus* plus Kannemeyeriiformes (Cox & Angielczyk, 2015; Kammerer, Fröbisch & Angielczyk, 2013; Kammerer & Smith, 2017). Recently it is found forming a stable clade with *Dinanomodon* and *Peramodon* (Angielczyk, Hancox & Nabavizadeh, 2017), and *Daptocephalus* (Angielczyk & Kammerer, 2017; Kammerer, 2019; Kammerer et al., 2019; Liu, 2020; Olivier et al., 2019).

To test the phylogenetic position of *Turfanodon jiufengensis*, it was coded based on the available specimens for the character list of Kammerer (2019), except for character 56 (discrete-state character 33) in which a new state (nasals without a median suture) is added (Supplemental Data 1). *Turfanodon bogdaensis* was also recoded based on two specimens (IVPP V 3241 and IGCAGS 296). The codings were added to the matrix of Liu (2020). The final data set consists of 112 operational taxonomic units (OTUs) and 197 characters (23 continuous and 174 discrete-state) (Supplemental Data 2). Continuous characters were treated as additive, and eight discrete-state characters were treated as ordered (characters 56, 58, 61, 79, 140, 150, 151 and 166). The data were analyzed using parsimony in TNT v1.5 (Goloboff & Catalano, 2016) using New Technology search parameters, starting at level 65 and forced to find the shortest tree at least 50 times checking every three hits. Symmetric resampling values were calculated based on 10,000 replicates.

**Figure 22:**
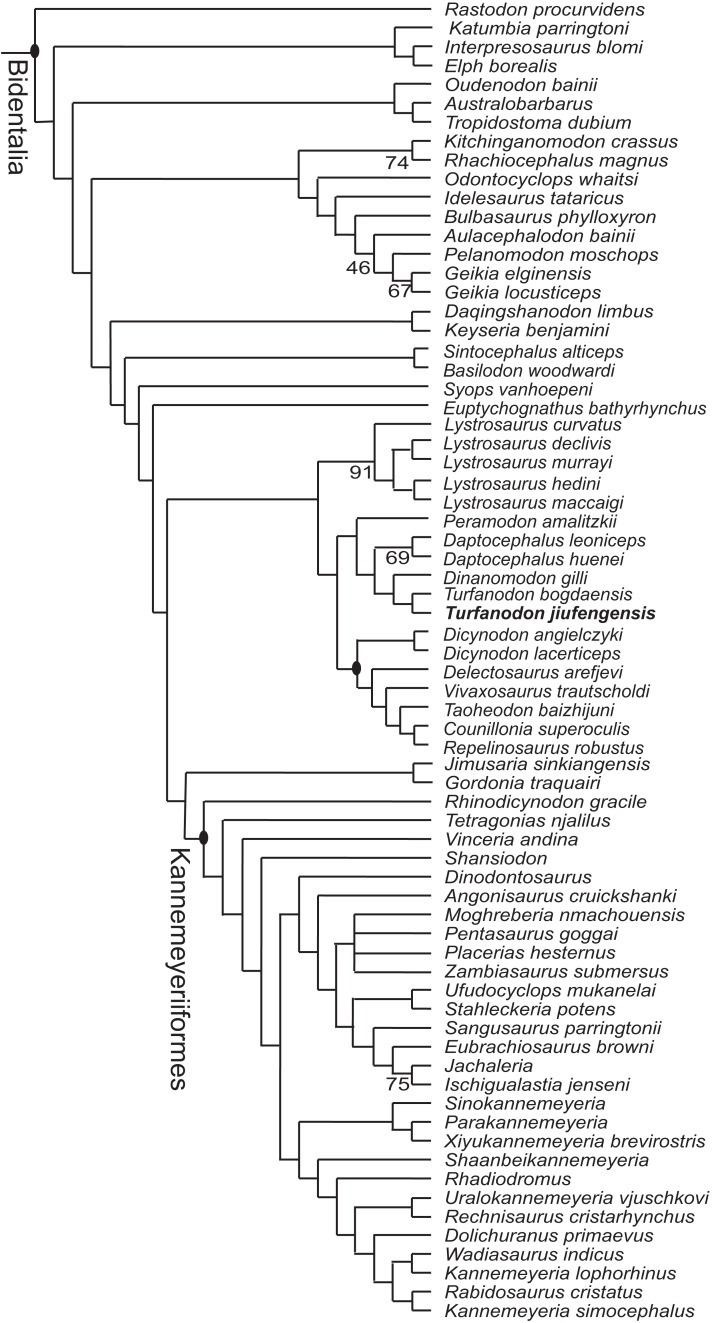
Phylogeny of Bidentalia. *Turfanodon jiufengensis* in bold. Numbers at nodes represent symmetric resampling values >45.

Three most parsimonious trees of length 1,213.086 were recovered, and the phylogeny of Bidentalia is shown in Fig. 22. The general pattern is almost identical to figure 6 of Liu (2020) and figure 13 of Kammerer (2019): Cryptodontia is limited to a clade including three genera (*Oudenodon*, *Australobarbarus*, and *Tropidostoma*), and the ‘core-*Dicynodon*’ clade of Liu (2020) is present. One distinct result here is the basalmost position of *L. curvatus* within *Lystrosaurus*, keeping with the traditional, pre-cladistic view (Cluver, 1971). A close relationship between the two species of *Turfanodon* is confirmed. They share synapomorphies such as the anterior tip of snout being squared off and the nasal bosses present as paired swellings near the posterodorsal margin of external nares.

### Postcranial features provided by complete specimens

Dicynodontoidea was one of the most successful dicynodont clades during the Permian. At present, there are nearly 20 valid species of Permian dicynodontoids (not including the Permo-Triassic boundary crosser *Lystrosaurus*). They are the most common tetrapods in Lopingian terrestrial assemblages around the world, for example, in the *Daptocephalus* Assemblage Zone of South Africa (Viglietti et al., 2016). Although many dicynodontoid species are known from abundant materials, they are generally only represented by skulls, and postcranial remains are rare, even for common genera such as *Dicynodon*. Up until now, postcranial characters could be coded in recent matrices only for *Vivaxosaurus trautscholdi*, *Peramodon amalitzkii*, *Gordonia traquairi*, and *Daptocephalus leoniceps* among the non-*Lystrosaurus* Permian dicynodontoids (Kammerer, 2019; Kammerer et al., 2019; Olivier et al., 2019; Liu, 2020). One Zambian specimen, NHMUK PV R37005 (formerly ‘TSK 14’), is a nearly complete skeleton that was initially referred to the species *Dicynodon trigonocephalus* (King, 1981a). This specimen was later referred to ‘*Dicynodon*’ *huenei* and used to code that taxon in most previous matrices (Kammerer, Angielczyk & Fröbisch, 2011; Angielczyk et al., 2014), but has recently been argued to represent a distinct species (Kammerer, 2019). IVPP V 26038 represents another nearly complete skeleton and the first such discovery from China.

This specimen provides rare information on the composition of a complete vertebral column in a dicynodont. Nearly complete vertebral columns are more frequently preserved in small-bodied anomodont species, for example, *Suminia*, *Pristerodon*, *Cistecephalus*, *Robertia*, *Diictodon*, and *Eosimops*; but they are rare in larger species, with only a few Permian representatives reported in the literature: *Endothiodon*, *Dicynodontoides*, ‘*Aulacocephalodon peavoti*’, *Peramodon*, and the Zambian *‘Dicynodon trigonocephalus*’ (Table 2). Based on these specimens, the cervical number is 6 in most anomodont species (as in basal synapsids; Müller et al., 2010), but five in *Cistecephalus* and *Endothiodon* (Cluver, 1978; Maharaj, Chinsamy & Smith, 2019) (though these records need to be confirmed by more specimens). The cervical number increased to 7 in some Triassic kannemeyeriiforms such as *Sinokannemeyeria* and *Ischigualastia* (Camp & Welles, 1956; Sun, 1963). The sacral number increased to 5 near the origin of Bidentalia, and there is a correlation between increased sacral count and larger body size (Griffin & Angielczyk, 2019). Most dicynodontoids have five sacrals, but some have 6 or even 7 sacrals (Table 2). The caudal numbers are only known in a few species, but most have 14 or 15 caudals except for the unusual, probably arboreal basal anomodont *Suminia* (>52) (Fröbisch & Reisz, 2011), *Lystrosaurus murrayi* (10) (Ray, 2006), and *Ischigualastia* (~6) (Camp & Welles, 1956). The dorsal vertebral count in dicynodonts is generally not greater than 21, for example, 20 in *L*. *murrayi*, *L. hedini*, and ‘*Dicynodon trigonocephalus*’ (Table 2), but in *Peramodon*, *Turfanodon*, and *Placerias* the dorsal number is notably higher.

In IVPP V 26038, steeply inclined zygapophyses and expansion of the sacrum suggested the stiffening of the trunk and reduction in the lateral movement of the vertebral column (Ray, 2006). Its scapula has a notch on ventral margin, showing that the coracoid foramen is bordered by both the scapula and procoracoid as in *Lystrosaurus hedini* (Young, 1935). The coracoid plate is reduced relative to the scapula, differing from primitive dicynodonts such as *Eodicynodon* (Rubidge, King & Hancox, 1994) and *Diictodon* (Ray & Chinsamy, 2003). Although incomplete, the ilium has a long preacetabular process and a short postacetabular iliac process. The acetabulum had a lateral orientation. The deltopectoral crest is about half length of the humerus, similar to Triassic kannemeyeriiforms but greater than in most Permian dicynodonts (Ray, 2006). The femur is similar to that of *Lystrosaurus* in having a narrow shaft and moderately-developed head (Ray, 2006; Young, 1935). The femoral midshaft cross-section has a ratio of 1.5, lying between *Lystrosaurus* and Triassic dicynodonts (Ray, 2006).

The right humerus is not fully prepared in the holotype, but the circumference of its shaft can be estimated as ~110 mm. The left femoral circumference is 100 mm. Based on these data, a body mass of the holotype of ~190 kg can be calculated using the equation: logBM = 2.749 · logC_H+F_ − 1.104 (Campione & Evans, 2012).

### Tetrapod fauna of the Naobaogou Formation: paleobiogeographic implications and biostratigraphic correlation

The Naobaogou Formation has produced dicynodonts (*Daqingshanodon limbus*, *Turfanodon jiufengensis*, cf. *Jimusaria*) (Liu, 2019; Zhu, 1989), the captorhinid *Gansurhinus qingtoushanensis* (IVPP V 12026) (Li & Cheng, 1997; Reisz et al., 2011), the pareiasaur *Elginia wuyongae* (Liu & Bever Gabriel, 2018), and three therocephalians (*Shiguaignathus wangi, Jiufengia jiai, Caodeyao liuyufengi*) (Liu & Abdala, 2017a, 2019, 2020) (Table 3). In Xinjiang, the Guodikeng Formation has produced dicynodonts (*Jimusaria sinkianensis, Turfanodon bogdaensis, Diictodon feliceps*, *Lystrosaurus hedini*, and *Lystrosaurus youngi*), the therocephalian *Dalongkoua fuae*, and bystrowianid chroniosuchians (Li, Wu & Zhang, 2008; Liu & Abdala, 2017b). They share *Turfanodon* and possibly *Jimusaria* as the common genera. The Naobaogou Formation further shares *Gansurhinus* with the Dashankou Fauna (Qingtoushan Formation) of Gansu and *Elginia* with the Cutties Hillock Sandstone of Scotland (Liu & Bever Gabriel, 2018).

**Table 3:** Distribution of catalogued specimens from the Naobaogou Formation.

IVPP V	Taxon	Member	Position
26038	*Turfanodon jiufengensis*	III	U
23297	*Shiguaignathus wangi*	III	L
23878	cf. *Daqingshanodon*	III	L
26036	Bidentalia indet.	III	L
23877	*Jiufengia jiai*	III	B
26035	cf. *Jimusaria*	II	M-U
26037	*Daqingshanodon*	II	M
23880	Bidentalia indet.	II	M
23298	*Caodeyao liuyufengi*	II	M
23880	*Turfanodon jiufengensis*	II	M
23875	*Elginia wuyongae*	II	L
12026	*Gansurhinus qingtoushanensis*	I	M
7940	*Daqingshanodon limbus*	I	L
26034	cf. *Jimusaria*	I	L

**Note:**

B, base; L, lower; M, middle; U, upper.

During the late Permian, dicynodonts were widely distributed from South Africa to the Junggar Basin, Xinjiang, but tropical records were known only from Scotland, Laos, and the Ordos Basin (Bernardi et al., 2017; Fröbisch, 2009). *Turfanodon bogdaensis* was distributed in a temperate zone, with a paleolatitude higher than N30°; whereas *T. jiufengensis* occurred in a tropical zone, with a paleolatitude of ~N20° (Bernardi et al., 2017; Huang et al., 2018; Li et al., 2003). Thus, *Turfanodon* is the first known dicynodont genus distributed both in tropical and temperate zones. Among other Permian amniotes, the members of the pareiasaur subclade Elginiidae were known mainly in tropics (Scotland, North China, Morocco), except *Obirkovia* from the temperate zone (South Urals).

The Naobaogou dicynodonts are closely related to *Keyseria* and *Dinanomodon* from South Africa, indicating a rough correlation with the *Daptocephalus* Assemblage Zone. *Elginia wuyongae* is closely related to *E. mirabilis* from the Cutties Hillock Sandstone and *Obirkovia* from the Sokolki Fauna and the Vyazniki Fauna (Bulanov & Yashina, 2005; Liu & Bever Gabriel, 2018). The therocephalians are closely related to Russian *Annatherapsidus* and *Purlovia*, from the Sokolki Fauna and the Vyazniki Fauna (Liu & Abdala, 2017a, 2019, 2020). *Lystrosaurus* is reported from upper *Daptocephalus* AZ, the Astashikha Fauna, and the Guodikeng Formation, but is absent from the Naobaogou Formation and Sunjiagou Formation. One possibility is that the upper boundary of the Naobaogou Formation is still younger than Astashikha Fauna, and *Lystrosaurus* appeared in this region later. However, its fossils are absent from the overlying Laowopo Formation, so it is possible local absence of *Lystrosaurus* in the Ordos Basin is genuine. Based on these comparisons, the Naobaogou Formation would be roughly 255—252 Ma in age (Liu, 2018).

## Supplemental Information

10.7717/peerj.10854/supp-1 Supplemental Information 1 Character list.

10.7717/peerj.10854/supp-2 Supplemental Information 2 Matrix. This file is using software TNT 1.5.

10.7717/peerj.10854/supp-3 Supplemental Information 3 Specimen repository data.

## Additional Information and Declarations

### Data Availability

The following information was supplied regarding data availability: The character and code are available in Supplemental Files. All five new specimens described in this manuscript are stored in IVPP (Institute of Vertebrate Paleontology and Paleoanthropology, Chinese Academy of Sciences, Beijing) with the following accession numbers: - IVPP V 23299, crushed skull with lower jaw - IVPP V 23879, a well-112 preserved and thoroughly-prepared snout; - IVPP V 23880, an incomplete small skull - IVPP V 26035, a three-dimensionally preserved skull, 7 vertebrae, and incomplete right forelimb - IVPP V 26038, a relatively complete skeleton, including incomplete skull and lower jaw.

### New Species Registration

The following information was supplied regarding the registration of a newly described species: Publication LSID: urn:lsid:zoobank.org:pub:72E571BB-4982-4294-886C-4700F9596AA7. *Turfanodon jiufengensis* sp. nov. LSID: urn:lsid:zoobank.org:act:D1EC2845-C8D1-47CD-BA4A-017DF8F87E85E85.
