# Supplementary material for: The tetrapod fauna of the upper Permian Naobaogou Formation of China: 6. Turfanodon jiufengensis sp. nov. (Dicynodontia)
Source: PeerJ. 2021 Feb 17;9:e10854. doi: 10.7717/peerj.10854 (PMC7896508; doi:10.7717/peerj.10854)
Supplement: Supplemental Information 3 [file peerj-09-10854-s003.docx]

Repository data

All five new specimens described in this manuscript are stored in IVPP (Institute of Vertebrate Paleontology and Paleoanthropology, Chinese Academy of Sciences, Beijing) with the following accession numbers:

IVPP V 23299, crushed skull with lower jaw

IVPP V 23879, a well-112 preserved and thoroughly-prepared snout;

IVPP V 23880, an incomplete small skull

IVPP V 26035, a three-dimensionally preserved skull, 7 vertebrae, and incomplete right forelimb

IVPP V 26038, a relatively complete skeleton, including incomplete skull and lower jaw
